# Guizhou Green Tea Metabolomics Analysis: Volatile Metabolites Based on HS‐GC‐IMS and UPLC‐Q‐TOF‐MSMS and Quality Evaluation

**DOI:** 10.1155/ijfo/1031648

**Published:** 2026-05-18

**Authors:** Hui-Xiong Zhong, Meng-Ying Wu, Fu-Xiang Li, Xiang-Yu Chen, Meng-Yuan Jiang, Mei-Ting Zhang, An-Qi Li, Zi-Teng Yu, Wei Liu, Ke-Ke Cheng

**Affiliations:** ^1^ Guangdong Provincial Key Laboratory of Distributed Energy Systems, Dongguan University of Technology, Dongguan, China, dgut.edu.cn; ^2^ China-Latin America Joint Laboratory for Clean Energy and Climate Change, School of Chemical Engineering and Energy Technology, Dongguan University of Technology, Dongguan, China, dgut.edu.cn; ^3^ Dongguan University of Technology Analytical and Testing Center, Dongguan, China

**Keywords:** metabolomic analysis, quality assessment, rOAVs, tea, VOCs

## Abstract

This study identifies metabolite profiles associated with aroma variations among three Guizhou green teas (Meitan Cuiya, Meitan Maofeng, and Duyun Maojian) using integrated HS‐GC‐IMS and UPLC‐Q‐TOF‐MSMS analysis. We identified 57 volatile organic compounds and 159 phenolic compounds, with 22 compounds exhibiting relative odor activity values (rOAVs) ≥ 1. A partial least squares regression (PLSR) model was developed to establish quantitative relationships between metabolite concentrations and electronic tongue sensory parameters, demonstrating predictive capability (*R* ^2^ *Y* = 0.89, *Q* ^2^ = 0.82). Variable importance in projection (VIP) analysis identified heptanal, butanol, and pentanol as major contributors to sensory variance. Significant correlations were observed between specific metabolites and sensory attributes (e.g., heptanal‐green perception: *r* = 0.82, *p* = 0.003). Unique metabolite combinations were identified for each tea variety: 2‐furanmethanol acetate for Meitan Cuiya, propanol for Meitan Maofeng, and myrcene‐D/M for Duyun Maojian. The OPLS‐DA model (*R* ^2^ *Y* = 99.3*%*, *Q* ^2^ = 98.2*%*) provides a classification tool for tea differentiation. Absolute quantification was achieved for 22 key aroma compounds and 15 major phenolics with complete method validation. These findings identify metabolite profiles associated with aroma variations among Guizhou green teas and provide candidate markers for quality assessment.

## 1. Introduction

Derived from the tender leaves of the *Camellia sinensis* (L.) O. Kuntze plant, tea ranks among the most widely consumed beverages on a global scale [1]. It is systematically classified into six principal categories according to the degree of fermentation: black tea, yellow tea, green tea, dark tea, white tea, and oolong tea [2]. Distinctive taste profiles are characteristic of the various tea types. The comprehensive assessment and evaluation of tea products predominantly concentrate on quality determinants, including appearance, color, aroma, and taste, all of which are grounded in sensory attributes [3]. Tea aroma serves as a critical determinant of tea quality and constitutes one of the primary factors associated with attracting consumers and cultivating brand loyalty. It holds significant importance in evaluating the overall quality of green tea and functions as an effective methodology for tracing the geographical origin of tea [4]. Tea aroma is constituted by a multitude of volatile compounds present at diverse concentrations [5]. The volatile compounds associated with tea exhibit considerable complexity, with over 700 distinct aroma compounds having been identified, yet they typically exist at trace concentrations (approximately 1 g/kg in total) [6]. Furthermore, it is predominantly the key odorants that direct sensory perception and contribute substantively to the overall aroma [7]. Green tea is distinguished by its unique and agreeable aroma quality, which is widely appreciated by tea consumers. The foundational nature of tea aroma encompasses a diverse assemblage of volatile organic compounds (VOCs) occurring at varying concentrations. The distribution of these constituents within the tea infusion, their relative proportions, and their interplay collectively culminate in a holistic aroma profile [8].

Prior research has established that amino acids and polyphenols constitute significant nutritional and bioactive constituents in green tea [9]. This includes the nutritional significance of important amino acids and the pharmacological actions of theanine and *γ*‐aminobutyric acid (GABA). Theanine, a principal amino acid uniquely present in green tea, has been demonstrated to decrease levels of norepinephrine and serotonin in the brain, lower blood pressure, and exert neuroprotective and cognitive‐enhancing effects [10–12]. GABA serves as a crucial inhibitory neurotransmitter within the mammalian central nervous system and is recognized for its antihypertensive properties [13]. The polyphenols derived from tea, commonly referred to as tea polyphenols, primarily consist of catechins and their derivatives, accounting for 50%–60% of the total polyphenolic mass in tea [14, 15]. Tea polyphenols exhibit substantial physiological activity and confer a multitude of health benefits, finding application in the development of antiviral agents, antitumor, diabetes, obesity, and cardiovascular health therapeutics [16]. The three green tea varieties selected for this investigation represent prominent teas from Duyun City and Meitan City, notably including DYMJ and MTCY, which rank among Guizhou’s most renowned teas. Furthermore, these selections reflect the cultural and economic significance of their respective production regions and are esteemed local specialties characterized by a long history of traditional craftsmanship.

In the realm of volatile food component detection, various instrumental methods present distinct trade‐offs in terms of speed, sensitivity, and ease of operation. Gas chromatography–ion mobility spectrometry (GC‐IMS) stands out as a significant technique in the analysis of food flavor and quality, encompassing a unique set of advantages and challenges [17, 18]. The olfactory characteristics of tea are profoundly influenced by its volatile constituents. Advanced analytical methodologies, such as gas chromatography–mass spectrometry (GC‐MS), when coupled with sample preparation techniques like headspace solid‐phase microextraction (HS‐SPME), simultaneous distillation extraction (SDE), stir bar sorptive extraction (SBSE), and solvent‐assisted flavor evaporation (SAFE), have facilitated the identification of a wide array of volatile compounds. The intricate blend of these volatiles, along with their respective concentrations, exhibits substantial heterogeneity among various tea varieties, thereby endowing each with its unique aromatic profile. However, GC‐MS is hindered by limitations such as slow analysis speed, complex pretreatment procedures, and sensitivity constraints. Fortunately, GC‐IMS offers a rapid analysis speed, simplified pretreatment, low detection limits, and high detection sensitivity, thereby compensating for some of the deficiencies of GC‐MS and providing significant advantages in the detection of trace substances [7, 19]. Besides, for the precise qualitative analysis of amino acids and polyphenolic compounds, this study utilized ultra‐performance liquid chromatography‐quadrupole time of flight tandem mass spectrometry (UPLC‐Q‐TOF‐MSMS technology. Compared with traditional amino acid analyzers, UPLC‐Q‐TOF‐MSMS) technology boasts superior sensitivity, detection capability, and powerful structural identification capabilities, as well as extremely high mass accuracy [20].

The objective of this research is to precisely and effectively identify and differentiate the aroma compounds, amino acids, and polyphenolic compounds present in green tea. By integrating the analysis of aroma profiles and relative odor activity values (rOAVs), we aim to explore the impact of VOC characteristics and aroma quality within the same tea variety from various regions and to pinpoint the specific components associated with the aroma quality variations among the three tea types. To thoroughly assess tea flavor, we employed multivariate statistical analysis techniques, including principal component analysis (PCA) and orthogonal partial least squares discriminant analysis (OPLS‐DA), to categorize the flavor substances in the three green tea samples. This is the pioneering application of combining headspace–gas chromatography–ion mobility spectrometry (HS‐GC‐IMS) and UPLC‐Q‐TOF‐MSMS technologies in the evaluation of Guizhou teas, with the intention of deepening our understanding of the characteristic marker compounds, amino acids, and polyphenolic compounds that define the flavor of different green tea samples. This study offers both a theoretical foundation and practical insights for the research on tea quality.

While regional comparison forms the foundational framework of this study, our work transcends descriptive geographical differentiation to quantitatively resolve three critical knowledge gaps within tea metabolomics: (1) Most existing studies merely report VOCs as qualitative lists, failing to establish quantitative linkages with sensory attributes. We address this limitation by integrating rOAV analysis electronic tongue data via partial least squares regression (PLSR) modeling, thereby establishing a predictive framework for aroma–sensory correlations. (2) The specific quantitative impact of individual metabolites on overall tea quality remains elusive. Our methodology uniquely identifies and quantifies the statistical contributions of key aroma compounds to specific sensory attributes through correlation analysis and variable importance in projection (VIP) scoring. (3) Robust tools for rapid regional tea quality authentication are scarce. We develop and validate a robust OPLS‐DA classification model, which exhibits high predictive efficacy for tea authenticity testing.

This study adopts regional comparison as its basis and surmounts the descriptive limitations inherent in traditional tea metabolomics. By employing PLSR, VIP scoring, and OPLS‐DA models, we quantitatively characterize the aroma–sensory relationship, elucidate the contributions of key metabolites to tea quality, and establish a robust model for the efficient authentication of geographical origin, thereby addressing three major research gaps in this field.

## 2. Experimental Section

### 2.1. Reagents and Standards

We obtained three different categories of green tea samples from local tea companies in Guizhou, namely, Guizhou Meitan Crispy Bud, Guizhou Meitan Maofeng, and Guizhou Duyun Maojian. All tea leaves were harvested during the same period and processed under similar conditions. The sources of these three green tea samples are detailed in Table S1. All green tea samples were stored in aluminum foil bags to avoid light and stored at −20°C for further analysis. Before detection, 3 g of each tea sample was weighed and soaked in 100 mL of boiling water for 5 min, and 5 mL was taken for detection. The amino acid standard sample, including o‐phosphoethanolamine (98.0%), aspartic acid (98.0%), threonine (98.0%), serine (98.0%), asparagine (98.0%), glutamic acid (98.0%), sarcosine (98.0%), theanine (98.0%), alpha‐aminoadipic acid (98.0%), proline (98.0%), valine (98.0%), and lysine (98.0%), was purchased from Jiaruisi Technology (Dongguan, China).

### 2.2. HS‐GC‐IMS Detection and Analysis

The methodology implemented was an enhanced version of the procedures described by Yang et al. in their 2018 and 2017 publications. The separation of volatile constituents was achieved using an MXT‐WAX‐1 organic column (15 m in length and 0.53 mm in internal diameter) sourced from Shimadzu Research Laboratory Co., Ltd., Shanghai, China. The chromatographic analysis method employed is consistent with that utilized by Lu et al. Samples (0.5000 ± 0.050 g) were weighed into 20 mL headspace flasks, mixed with 3 mL of 3 mol/L NaCl solution and 1 *μ*L of 100 ng/*μ*L ethyl decanoate n‐hexane solution (internal standard). This procedure entails incubating at 60°C with a rotation speed of 500 rpm for a duration of 15 min. Subsequently, 0.5 mL of aroma is transferred from the headspace vial and automatically injected into the injection port utilizing a syringe, while maintaining a constant temperature of 85°C throughout the entire process. The chromatographic analysis was carried out for 26 min, with an injection volume of 500 *μ*L and a drift gas flow rate of N_2_ set at 150 mL/min. The elution profile was meticulously designed: a constant flow rate of 2 mL/min for the initial 2 min, increasing to 10 mL/min between 2 and 10 min, further escalating to 100 mL/min from 10 to 20 min, and reaching a peak of 150 mL/min from 20 to 25 min. Triplicate GC‐IMS analyses were conducted to ensure methodological reproducibility. Retention index (RI) was determined using a calibration mixture of n‐ketones (C4–C9). Compound identification was accomplished by comparing RI values and DTs with the NIST database and the proprietary GC × IMS library search software accompanying the IMS database.

For the compounds with high rOAV values (rOAV ≥ 1), absolute quantification was performed using external calibration curves with authentic standards. Standard preparation: Authentic standards were purchased from Sigma‐Aldrich (St. Louis, Missouri, United States) and Jiaruisi Technology (Dongguan, China). Stock solutions (1000 *μ*g/mL) were prepared in n‐hexane and stored at −20°C. Working solutions were prepared by serial dilution to establish calibration curves. Five to seven concentration levels were used for each compound. Calibration curves were constructed by plotting peak area ratios (analyte/internal standard) against concentration ratios. Linear regression was applied with a weighting factor of 1/*x*. Acceptance criterion: correlation coefficient *R* ^2^ > 0.99. Detailed validation parameters are provided in Table S2.

### 2.3. Quantitative Methods

The raw data from GC‐MS assays were processed utilizing the unknown analysis feature in the MassHunter workstation. Compounds exhibiting a frequency of occurrence of at least 2/3 in each sample group and at least 3/4 in the quality control (QC) samples were selected for further analysis. Among these, substances with mass spectral matches of 70 or higher and RI values of 10 or less were characterized. The relative content of these compounds was then calculated using a formula corrected by the internal standard of ethyl decanoate.

Relative content=peak area of compoundspeak area of internal standard×internal standard quality.

### 2.4. UPLC‐Q‐TOF‐MSMS Detection and Analysis

The quantification of nonvolatile constituents, including amino acids and polyphenolic compounds, was conducted using the UPLC‐Q‐TOF‐MSMS system (AB Sciex Co., Ltd., Framingham, Massachusetts, United States). This process adhered to the methodology outlined by Kang et al. [21], with certain modifications applied to the original procedure. The tea extracts were subjected to centrifugation at 1948 g for a duration of 10 min, followed by filtration through a 0.22‐*μ*m porosity filter. Subsequently, the prepared samples were introduced into an Agilent HC‐C18 analytical column (4.6 × 150 mm and 5 *μ*m particle size) for separation, utilizing a binary mobile phase system comprising 0.1% formic acid in water (Solvent A) and acetonitrile (Solvent B). For amino acid profiling, the instrument was operated in positive ion mode with an injection volume of 3 *μ*L. For polyphenol profiling, the instrument was operated in negative ion mode, with an injection volume of 5 *μ*L, a flow rate of 0.25 mL/min, and a column temperature of 50°C. The chromatographic parameters were optimized as follows: flow rate, 0.3 mL/min; column temperature, 35°C; and a gradient elution profile was applied, transitioning as follows: 0–2 min, 99% A; 2–3.25 min, 99%–95% A; 3.25–4.25 min, 95% A; 4.25–7.75 min, 95%–45% A; 7.75–9.75 min, 45%–10% A; 9.75–11.75 min, 10% A; 11.75–12 min, 10%–99% A; and 12–15 min, 99% A. The instrumental settings for the UPLC‐Q‐TOF/MS analysis were as follows: ESI voltage, 3.5 kV; capillary temperature, 320°C; mass range, 50–1000 m/z; mass resolution, 70,000 FWHM; sheath gas, 40 Arb; curtain gas, 10 Arb; and the temperature of the heated nebulizer gas was set to 350°C, in accordance with the specifications provided by Wu et al. [22]. Using the XCMS software, raw mass spectrometry data was imported and subjected to peak alignment and extraction. This process facilitated the identification of molecular ion peaks and isotope peaks, which were then utilized to infer potential molecular formulas. The resultant secondary fragment spectra were compared against the mz‐Cloud and mz‐Vault databases. A match was considered valid if it achieved a score of 80 or higher. Furthermore, the mass accuracy for both primary and secondary ions was required not to exceed 5 ppm, and the peak area had to be at least 80,000 to be considered a qualified match. Ultimately, compounds were analyzed and identified by correlating the filtered ions with compound information in the databases and relevant scientific literature.

Amino acids were quantified using the external standard method with authentic standards. The calibration curves were established using amino acid standard mixtures at five concentration levels. Method validation including linearity, LOD, LOQ, recovery, and precision was performed. Detailed validation parameters are provided in Table S2. A tiered quantification approach was applied for phenolic compounds: 15 major phenolic compounds with available authentic standards were quantified using external calibration curves. Standards included gallic acid, chlorogenic acid, caffeic acid, vanillic acid, catechin, epicatechin, epicatechin gallate, quercetin, kaempferol, rutin, myricetin, hyperoside, and others. Full method validation was performed (Tables S3 and S4). For compounds without authentic standards, semiquantification was performed using class‐appropriate internal standards. This approach is consistent with current metabolomics practices and enables comprehensive profiling while acknowledging quantitative limitations.

### 2.5. Multivariate Statistical Analysis

PCA was applied in order to gain a preliminary understanding of the overall metabolite differences between the samples in each group and the magnitude of variability between the samples within the groups. The partial least squares discriminant analysis (PLS‐DA) was utilized to extract the VIP value of each comparison group to screen for differential volatile metabolites. The rOAV used to identify key volatile compounds from tea was calculated according to the formula [23].

## 3. Results and Discussion

### 3.1. Comparison of Topographic Maps of Various Green Tea Samples Using HS‐GC‐IMS

**Figure 1:**
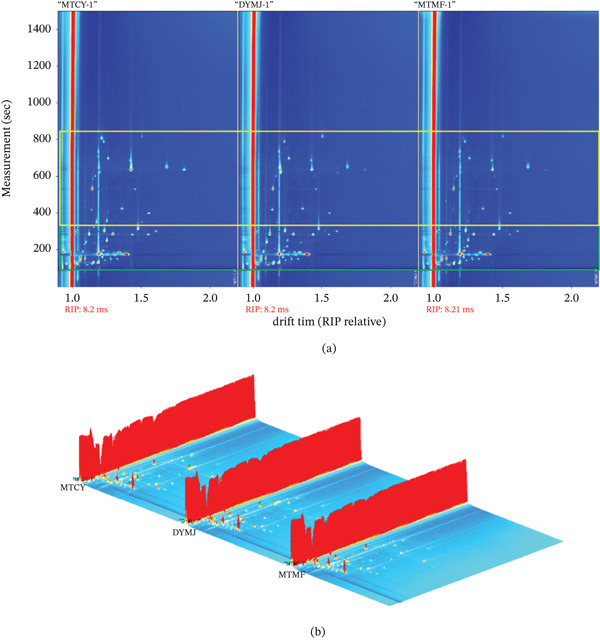
(a) 2D and (b) 3D topographic plots of volatiles in three green tea samples.

For the qualitative analysis of various green tea samples, the HS‐GC‐IMS method was utilized, employing a two‐dimensional (2D) separation technique for flavor substances [24]. Comprehensive IMS data was acquired to identify VOCs and discriminate between variations among the green tea samples. HS‐GC‐IMS 2D/3D topographic images serve to depict the overall fingerprint profiles of diverse volatile compounds present in the tea samples [25, 26]. The topographic maps derived from HS‐GC‐IMS results for different green tea samples are presented in Figure 1. The abscissa represents the IMS drift time, whereas the ordinate denotes the GC retention time. Following normalization, the reaction ion peak is located at 1.0, illustrated as a red vertical line, with the background depicted in blue. Each point on the topographic map corresponds to an individual flavor compound. The coloration of each point indicates the concentration of VOCs, wherein white signifies a lower concentration of the analyte components, and red indicates a higher concentration. The 3D spectrum of VOCs in the green tea samples is illustrated in Figure 1a, wherein elevated flavor concentrations are reflected by more intense red hues. Variations in peak volume allow for the assessment of volatile compound diversity. Figure 1b presents the 2D topographic maps of three green tea samples, encompassing a relative drift time range of 1.0–2.5 and a retention time range of 100–1700 s. As depicted in Figure 1b, attributable to categorical distinctions, both the content and types of volatiles within the designated yellow box exhibit an increasing trend. The majority of the key volatile components across the three green tea samples are contained within the green box, demonstrating notable differences in signal intensity for specific components.

**Figure 2:**
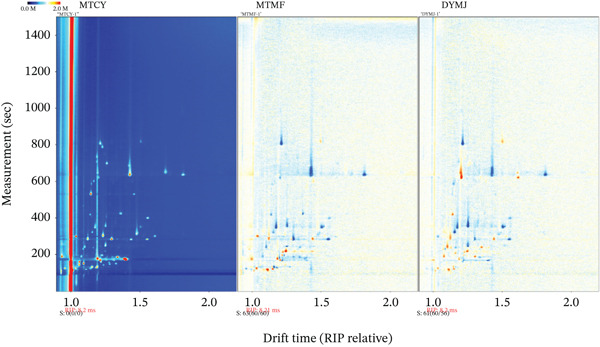
2D topographic plots (MTCY sample was selected as a reference).

To facilitate a more precise comparison of the variations among green tea samples, the topographic maps were subjected to analysis using the proprietary software of the HS‐GC‐IMS system. The MTCY spectrum was designated as the reference, and spectral values were subsequently subtracted from this reference point. As illustrated in Figure 2, white regions signify unchanged levels of VOCs postsubtraction, while red regions indicate concentrations of flavor substances that surpass the reference value. Conversely, blue regions denote concentrations of volatile substances that are lower than the reference value. Given that both MTMF and DYMJ samples are green teas, the predominant white background in the comparative model suggests minimal variation in the content of VOCs among the three green tea samples. Nevertheless, the presence of scattered red dots within the retention time range of 100–400 s indicates that discernible differences in volatile compounds still exist among the three samples, with all such red dots appearing in the spectra of MTMF and DYMJ. In conclusion, the unique species characteristics and growth environment of the green tea samples have positively influenced the accumulation of specific volatile compounds, rendering them key identifiers for species and origin differentiation. Consequently, the differentiation of volatile substances among samples can be effectively achieved through HS‐GC‐IMS technology and the corresponding visual topographic representations.

### 3.2. Identification of VOCs by Fingerprint Spectroscopy

**Figure 3:**
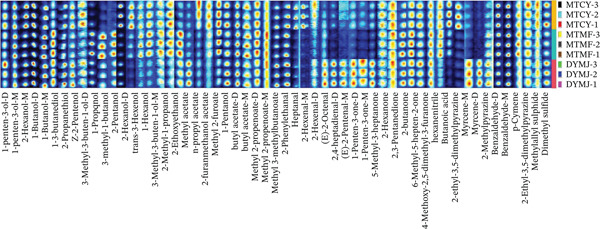
Global overview of the volatile identifier by HS‐GC‐IMS.

The integration of high‐resolution gas chromatography and highly sensitive ion mobility spectrometry facilitates the qualitative analysis of green tea with complex matrices, significantly reducing ion competition and cross‐interference. The underlying mechanisms and applications of HS‐GC‐IMS have been comprehensively elucidated in previous studies [7, 27]. The drift tube is equipped with a dedicated region for the ionization and separation of VOCs [28]. The green tea samples undergo initial heating and vaporization, followed by transportation to the ionization zone via nitrogen, where ionization occurs through an ion source [29]. This process generates various subions with distinct drift times in the separation region. The flavor concentration effects associated with the detection of multiple dimer signals in the ionization zone. Utilizing the HS‐GC‐IMS mechanism, different ketones, such as 2‐butanone, 2,3‐pentanedione, and 2‐hexanone, are employed for normalizing the calibration curves of retention time and RI. Figure 3 illustrates the selected characteristic volatiles from the topographic maps of three green teas, as retrieved from the IMS database. Table 1 provides detailed parameters of specific compounds and the qualitative analysis results of flavor substances. In the IMS drift time, monomers are denoted with the Suffix “M” in parentheses, whereas dimers are indicated by the Symbol “D.”

**Table 1:** Information of all volatile metabolites. “—” indicates “not detected” or “very low content.”

**Number**	**CAS**	**Compound Name**	**Category**	**Odor (** http://www.perflavory.com/search.php **)**	**Threshold (mg/mL)**	**Retention index**	**Concentration (ng/g)**						**rOAV**		
							**MTCY**	**sd**	**MTMF**	**sd**	**DYMJ**	**sd**	**MTCY**	**MTMF**	**DYMJ**

1	626‐93‐7	2‐Hexanol	Alcohols	Chemical winey, fruity, fatty, terpenic cauliflower	0.005	783.6	186.13	11.78	0.00	0.00	0.00	0.00	6.20	0.00	0.00
2	107‐88‐0	1,3‐Butanediol	Alcohols	‐	—	783.7	20.50	3.01	33.60	0.016	38.27	0.082	—	—	—
3	1576‐95‐0	(Z)‐2‐Pentenol	Alcohols	Green, phenol, nasturtium, ethereal, medicinal, aldehydic, cherry, narcissus, metallic, fruity	—	760	16.90	3.05	21.73	2.34	39.43	4.51	0.66	0.59	12.56
4	6032‐29‐7	2‐Pentanol	Alcohols	Mild, green, fusel oil, fermented	0.03	706.2	0.00	0.00	17.80	2.25	3.33	1.01	0.00	7.55	0.05
5	75‐33‐2	2‐Propanethiol	Alcohols	Strong ripe onion, gassy meaty, sulfurous	0.14	557.6	0.00	0.00	2.00	1.32	35.03	2.77	12.51	0.86	0.00
6	71‐23‐8	Propanol	Alcohols	Alcoholic, fermented, fusel musty	0.0028	524.6	0.00	0.00	13.75	2.02	0.00	0.00	0.00	8.66	0.00
7	71‐41‐0	Pentanol	Alcohols	Fusel oil, sweet, balsam	0.0015	750.4	2.20	1.39	30.20	2.16	42.85	12.37	0.12	20.13	28.57
8	111‐27‐3	Hexanol	Alcohols	Ethereal fusel oil, fruity, alcoholic, sweet green	0.04	860.2	0.00	0.00	11.23	0.93	6.00	0.00	0.00	15.88	0.32
9	928‐97‐2	trans‐3‐Hexenol	Alcohols	Green cortex privet, leafy floral petal, oily, earthy	0.35	847.4	35.93	3.06	139.47	18.32	249.63	2.65	0.10	0.40	13.47
10	78‐83‐1	2‐Methyl‐1‐propanol	Alcohols	Ethereal winey cortex	0.858	613	7.67	0.81	53.31	26.61	8.97	0.40	0.01	0.06	0.01
11	763‐32‐6	3‐Methyl‐3‐buten‐1‐ol	Alcohols	Sweet fruity	0.05	734.3	0.00	0.00	0.92	0.00	51.50	5.12	43.37	0.01	0.00
12	110‐80‐5	2‐Ethoxyethanol	Alcohols	—	—	726.4	20.56	2.55	33.60	2.07	38.27	3.33	—	—	—
13	123‐51‐3	3‐Methyl‐1‐butanol	Alcohols	Alcoholic, whiskey, banana	0.0008	712.8	7.67	0.81	130.23	7.36	8.97	0.40	0.01	18.25	0.06

**Number**	**CAS**	**Compound name**	**Category**	**Odor (** http://www.perflavory.com/search.php **)**	**Threshold (mg/mL)**	**Retention index**	**Relative concentration (ng/g)**						**rOAV**		
							**MTCY**	**sd**	**MTMF**	**sd**	**DYMJ**	**sd**	**MTCY**	**MTMF**	**DYMJ**

14	71‐36‐3	Butanol	Alcohols	Fusel oil, sweet, balsam, whiskey	9.562	655.7	88.55	3.28	8.00	3.36	100.73	9.58	19.01	18.59	18.25
15	616‐25‐1	1‐Penten‐3‐ol	Alcohols	Ethereal horseradish, green radish, chrysanthemum, vegetable, tropical fruity	0.051	667	10.37	0.85	33.43	1.58	8.94	0.40	0.10	0.40	0.71
16	123‐86‐4	Butyl acetate	Esters	Ethereal solvent, fruity, banana		797.2	8.67	0.38	40.30	3.84	24.27	6.27	0.52	0.33	0.22
17	96‐33‐3	Methyl‐2‐propenoate	Esters	—	—	608.3	10.26	2.57	20.66	4.25	0.00	0.00	—	—	—
18	611‐13‐2	Methyl‐2‐furoate	Esters	Fruity mushroom, fungus, tobacco, sweet	0.000027	973.9	0.00	0.00	17.10	1.70	35.03	1.85	0.00	0.79	13.67
19	109‐60‐4	n‐Propyl acetate	Esters	Celery, fruity, fusel, raspberry pear	0.01	699.4	47.00	3.08	5.87	0.42	0.00	0.00	19.38	0.88	0.00
20	556‐24‐1	Methyl 3‐methylbutanoate	Esters	Strong apple fruity, pineapple	0.011	773.3	50.13	2.47	163.10	8.70	68.90	1.37	1.12	1.47	1.19
21	79‐20‐9	Methyl acetate	Esters	Ether sweet fruity	0.05	538.4	10.40	1.30	44.61	0.83	0.00	0.00	0.09	8.93	0.00
22	623‐17‐6	2‐Furanmethanol acetate	Esters	Sweet, fruity, banana, horseradish	10	994	0.00	0.00	2.70	0.08	21.97	3.01	10.52	0.01	0.00
23	1576‐87‐0	(E)‐2‐Pentenal	Aldehydes	Pungent, green fruity, apple, orange, tomato	0.5	738.8	5.53	0.23	1.59	0.48	0.00	0.00	0.00	0.75	9.28
24	122‐78‐1	2‐Phenylethanal	Aldehydes	Green sweet, floral, hyacinth clover honey, cocoa	1.96	1032.4	0.00	0.00	7.91	0.89	72.80	1.27	0.00	0.05	19.38
25	5910‐85‐0	2,4‐Heptadienal	Aldehydes	Green pungent, fruity, spicy	0.004	1000.9	0.00	0.00	0.00	0.00	99.62		0.00	0.00	25.36
26	505‐57‐7	2‐Hexenal	Aldehydes	Green leafy, apple, vegetable	0.01	839.3	7.93	0.71	9.77	0.75	32.43	1.33	3.24	0.98	0.79

**Number**	**CAS**	**Compound name**	**Category**	**Odor (** http://www.perflavory.com/search.php **)**	**Threshold (mg/mL)**	**Retention index**	**Relative concentration (ng/g)**						**rOAV**		
							**MTCY**	**sd**	**MTMF**	**sd**	**DYMJ**	**sd**	**MTCY**	**MTMF**	**DYMJ**

27	111‐71‐7	Heptanal	Aldehydes	Fresh aldehydic, fatty green herbal, wine‐lee, ozone	15	897.8	73.27	3.52	51.67	3.16	72.80	1.47	19.38	13.67	19.26
28	2548‐87‐0	(E)‐2‐Octenal	Aldehydes	Fresh cucumber, fatty green, herbal banana, waxy green leaf	—	1049.7	47.00	3.08	49.67	4.30	113.80	3.93	—	—	—
29	110‐93‐0	6‐Methyl‐5‐hepten‐2‐one	Ketones	Citrus, green musty, lemongrass, apple	0.01	981	7.93	0.71	29.77	0.75	32.43	1.33	2.21	2.98	3.24
30	1629‐58‐9	1‐Penten‐3‐one	Ketones	Pungent, peppery mustard, garlic, onion	0.781	667.1	0.00	0.00	—	4.40	41.17	1.20	0.00	0.22	206.70
31	78‐93‐3	2‐Butanone	Ketones	Acetone‐like ethereal fruity camphor	0.058	583.3	5.03	0.47	7.10	0.82	7.43	1.89	—	—	—
32	541‐85‐5	5‐Methyl‐3‐heptanone	Ketones	Herbal sweet oily	—	947.8	6.53	0.32	27.47	1.85	2.55	0.07	2.22	1.36	1.88
33	4077‐47‐8	4‐Methoxy‐2,5‐dimethyl‐3(2H)‐furanone	Ketones	Sweet moldy mushroom, vegetable, potato, burnt sugar nut skin, wasabi, caramellic fruity brandy	—	1053.2	14.83	1.07	17.63	0.32	17.37	0.72	—	—	—
34	600‐14‐6	2,3‐Pentanedione	Ketones	Pungent sweet butter, creamy caramel, nutty cheese	—	684.6	0.00	0.00	9.57	0.40	0.00	0.00	—	—	—
35	591‐78‐6	2‐Hexanone	Ketones	Fruity, fungal, meaty, buttery	0.087	790.3	18.75	0.35	131.33	6.62	70.07	6.69	1.22	1.51	1.81
36	109‐08‐0	2‐Methyl pyrazine	Other	Nutty, cocoa, roasted chocolate, peanut, green	0.006	816.9	0.00	0.00	39.33	3.79	0.00	0.00	0.00	0.00	6.56
37	13925‐07‐0	2‐Ethyl‐3,5‐dimethylpyrazine	Other	Burnt almonds, roasted nuts, coffee	0.064	1081.4	17.00	0.00	16.53	1.59	9.45	0.49	0.20	0.19	0.11

**Number**	**CAS**	**Compound name**	**Category**	**Odor (** http://www.perflavory.com/search.php **)**	**Threshold (mg/mL)**	**Retention index**	**Relative concentration (ng/g)**						**rOAV**		
							**MTCY**	**sd**	**MTCY**	**sd**	**MTCY**	**sd**	**MTCY**	**sd**	**MTCY**

38	27043‐05‐6	2‐Ethyl‐3,5(6)‐dimethylpyrazine	Other	Burnt coffee, nutty roasted, woody	0.006	1090.9	133.33	6.62	18.75	0.35	70.07	6.96	1.51	0.22	0.81
39	75‐18‐3	Dimethyl sulfide	Ether	Sulfury, onion, sweet corn, vegetable, cabbage, tomato, green radish	0.064	536.6	40.27	3.52	87.83	2.53	52.37	8.86	1.63	0.37	1.82
40	10152‐76‐8	Methyl allyl sulfide	Ether	Alliaceous garlic, onion	0.781	688.2	0.00	0.00	9.57	4.60	0.00	0.00	0.00	0.00	0.00
41	99‐87‐6	p‐Cymene	Other	Fresh citrus, terpene woody spice	—	1038.1	8.37	1.25	11.90	0.70	8.93	0.40	—	—	—
42	123‐35‐3	Myrcene	Other	Peppery terpene, spicy balsam, plastic	0.01	988.3	—	4.45	2.40	0.00	375.75	5.49	0.86	0.85	37.27
43	100‐52‐7	Benzaldehyde	Aldehyde	Strong sharp, sweet bitter, almond cherry	—	946.2	6.53	0.4	—	0.2	—	0.05	1.22	1.56	2.99
44	107‐92‐6	Butanoic acid	Other	Sharp acetic, cheese, butter, fruit		815.9	76.20	3.19	5.24	2.73	16.33	5.00	24.13	0.66	0.89
45	628‐73‐9	Hexanenitrile	Other	—	—	878	0.90	0.00	2.40	0.00	1.45	0.07	—	—	—

The analysis of retention time and drift time in the HS‐GC‐IMS library, as detailed in Table 1, facilitated the detection of 57 VOCs across three distinct green tea samples, with all compounds being successfully identified. To delineate the flavor substances within these green tea samples, Figure 3 presents the fingerprint spectra of HS‐GC‐IMS volatile markers. Each column of fingerprints corresponds to a specific VOC, while each row represents the analytical outcomes for the respective green tea samples. Each sample was subjected to triplicate testing, labeled consistently with a designated name and numerical identifier. This method enabled the identification of distinctive volatile compounds in various green tea samples. For instance, the MTCY sample exhibited elevated concentrations of 2‐furanmethanol acetate [30, 31]. The MTMF sample, on the other hand, was characterized by volatiles such as hexanenitrile, 2‐butanone, and 2,3‐pentanedione, among others. The DYMJ sample displayed higher quantities of compounds like 2‐methylpyrazine, myrcene‐D, myrcene‐M, and more, in comparison to the other green tea samples [32]. Additionally, a comparison between the MTCY and MTMF samples revealed shared volatile compounds, including methylallyl sulfide and p‐cymene [33, 34]. Figure 3 illustrates the exceptionally high volatile signals for compounds such as methylallyl sulfide and 2‐ethyl‐3,5‐dimethylpyrazine. These characterized VOCs serve as visual indicators for distinguishing various green tea samples.

### 3.3. Analysis of Key Aroma Substances (rOAV ≥ 1) of MTCY, MTMF, and DYMJ

The majority of compounds with high rOAV values that significantly contribute to the aroma (rOAV ≥ 1) are also the primary odors of other green teas, including pentanol, 1‐butanol, methyl 3‐methylbutanoate, and heptanal [35] (Table 1). Pyrazines are nitrogen‐containing heterocyclic compounds characterized by their olfactory properties of baking, smoking, and earthy smells. These compounds exhibit low odor detection thresholds, for example, 2‐ethylpyrazine ranges from 0.1 to 10 ng/L. The sensory impact of pyrazines is influenced by their concentration and the human odor threshold [36]. Aldehyde‐containing oxygenated compounds are primarily associated with green, fruity, and oxidized aromas, and their sensory perception highly depends on structural changes [37]. For instance, in green tea, hexanal and (E)‐2‐hexenal are dominant odorants, with fresh, grassy, or cucumber‐like aromas detectable at thresholds of 0.5–1 *μ*g/L. (E)‐2,4‐Heptadienal aldehyde brings a unique “green pea” aroma. Esters, derivatives of carboxylic acids, are primarily associated with fruity, floral, or honey‐like sweetness, and their sensory contribution is significantly influenced by concentration and olfactory synergistic effects [38].

Furthermore, it is clear that heptanal was detected in all three green tea samples (MTCY: rOAV = 19.38, MTMF: rOAV = 13.67, and DYMJ: rOAV = 19.26). Based on its high rOAV values, heptanal is a candidate contributor to green or grassy aroma notes. Our correlation analysis showed a significant association between heptanal concentration and green perception (*r* = 0.82, *p* = 0.003). The potential contribution of heptanal to green tea aroma has been reported in literature [39], although actual sensory impact in the tea matrix requires validation. The alcohols identified through HS‐GC‐IMS were exclusively straight‐chain alcohols. 2‐Ethoxyethanol, detected in all samples, has been reported to exhibit fruity aroma characteristics reminiscent of ripe bananas and apples [40]. However, no corresponding rOAV values above 1 were calculated for this compound in the three green tea samples, suggesting that its potential contribution may be limited under the conditions studied. The volatile compound 1‐butanol, known for its sweet and fresh banana‐like aroma [41], was consistently detected in all three analyzed green tea samples (MTCY with an rOAV of 19.01, MTMF with 18.59, and DYMJ with 18.25). A few aromatic VOCs (rOAV > 1) played a significant role only in individual samples, imparting unique aroma characteristics to teas with varying levels of fermentation. In the MTCY sample, key aromatic VOCs such as 2‐hexanol, trans‐3‐hexenol, methyl 2‐furoate, and 2‐furanmethanol acetate were identified, with n‐propyl acetate and 2‐furanmethanol acetate being unique to this sample. These compounds contributed various aromas, including fruity, alcoholic, pear‐like, sweet, and spicy notes. Z‐2‐Pentanol was considered a key factor for the subtle changes in spicy, metallic, and cherry flavors in green tea samples [42].

Furthermore, 2‐pentanol, propanol, and pentanol are significant alcohols in the MTMF sample, with propanol being unique to this sample (Table 1). 2‐Pentanol (rOAV 7.55) is an alcohol compound that imparts a milk, green, chemical, petroleum, and fermented taste. Propanol (rOAV 8.66) is characterized by an alcoholic, fermented, fatty oil, and musty flavor. Pentanol (rOAV 20.13) is known for its fatty oil, sweet, and balsamic flavor. Among the key aromatic VOCs in the three tea samples, pentanol, butanol, 2‐hexanone, and benzaldehyde‐D have rOAV values greater than 1. Pentanol was detected only in the DYMJ (rOAV 28.57) and MTMF (rOAV 20.13) samples, contributing a fatty oil odor, sweetness, and balsamic flavor. Dimethyl sulfide, an ether compound with a sulfur, onion, sweet corn, and vegetable aroma, was found only in the DYMJ and MTCY samples, with rOAV values of 1.82 and 1.63, respectively. 1‐Propanol, a unique volatile marker in the MTMF sample, has an rOAV value of 8.66, characterized by subtle differences in alcohol, earthiness, and fruitiness reminiscent of apples and pears [43].

In the analysis of the three green tea samples, benzaldehyde, 5‐methyl‐3‐heptanone, 6‐methyl‐5‐hepten‐2‐one, heptanal, methyl 3‐methylbutanoate, and butanol were identified as key aromatic VOCs, all with rOAV values greater than 1. Benzaldehyde is an aldehyde compound associated with fermentation, fruitiness, greenness, and waxiness [44]. Additionally, 5‐methyl‐3‐heptanone and 6‐methyl‐5‐hepten‐2‐one are linked to citrus, greenness, mustiness, citronella, and apple characteristics [44]. The presence of heptanal adds a wine‐like flavor, while butanol imparts a whiskey‐like aroma.

### 3.4. Establishment of Multivariate Statistical Models for Green Tea Detection

**Figure 4:**
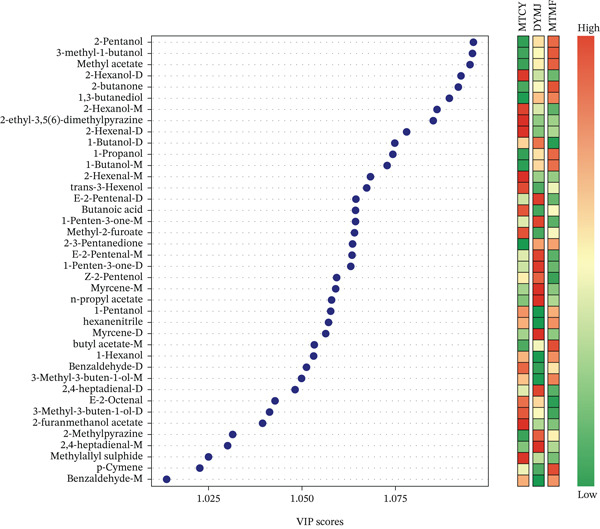
Differential volatile metabolites of each group were screened by PLS‐DA analysis. All 40 compounds with VIP > 1.

Using the PLS‐DA model, the VIP values of all volatiles in teas with different fermentation levels were calculated (Figure 4). Screening with VIP ≥ 1 found 40 common differential volatile metabolites in MTCY, DYMJ, and MTMF, which could potentially distinguish teas based on their production regions. Among them, 33 aromatic VOCs contributed to the variance in the aromatic quality at different fermentation levels. Twenty‐two major odor compounds (rOAV ≥ 1) distinguished the unique aromas of these three teas. These include six alcohols (2‐pentanol, 3‐methyl‐1‐butanol, 2‐hexanol, 1,3‐butanediol, 1‐butanol, and 1‐propanol), five other categories (carboxylic acids, alkenes, etc.), two aldehydes (2‐hexenal and E‐2‐pentenal), one ester (n‐propylacetate), and two ketones (1‐penten‐3‐one) with sweet, floral, fruity, citrus, rose, violet, lemon, apple, and melon aromas. Additionally, three alcohols (Z‐2‐pentanol, 1‐pentanol, and trans‐3‐hexenol), two esters (methyl‐2‐furoate and methyl acetate), and one ketone (2,3‐pentanedione, 2‐butanone) have woody, green, herbal, and spicy characteristics. Among the mentioned substances, 3‐methyl‐1‐butanol, 2‐hexanol, and 1,3‐butanediol (rOAV ≥ 1) have been reported as the main odor sources of chestnut‐like, floral, and fresh aromas in green tea [45].

**Figure 5:**
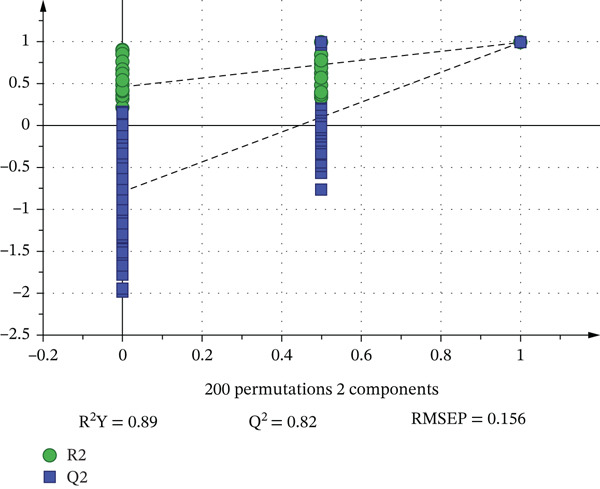
Establishment of a quantitative relationship model between metabolite profiles and sensory characteristics.

To examine the correlation model between the concentrations of VOCs and the sample groups, a supervised discriminant analysis statistical methodology must be established. The OPLS‐DA model was developed to differentiate the VOCs accountable for the quality variations in green tea samples [46, 47]. This OPLS‐DA model further enabled the visualization of high‐dimensional data [48, 49]. All VOCs underwent logarithmic transformation, with the detailed methodology documented in Text S1. As depicted in Figure 5, three distinct clustering categories are discernible through the OPLS‐DA model. The quadrant diagram provides a clear delineation of the differentiation observed in Figure 5. Specifically, MTMF samples are situated in the first quadrant, MTCY samples are positioned in the third quadrant, and the DYMJ sample is localized between the second and third quadrants. The computational results produced an *R* ^2^ *X* value of 85.9%, an *R* ^2^ *Y* value of 99.3%, and a predictive ability parameter (*Q* ^2^) of 98.2%, thereby confirming the excellent predictive efficacy of the OPLS‐DA model.

### 3.5. Quantitative Relationships Between Metabolites and Sensory Attributes

**Figure 6:**
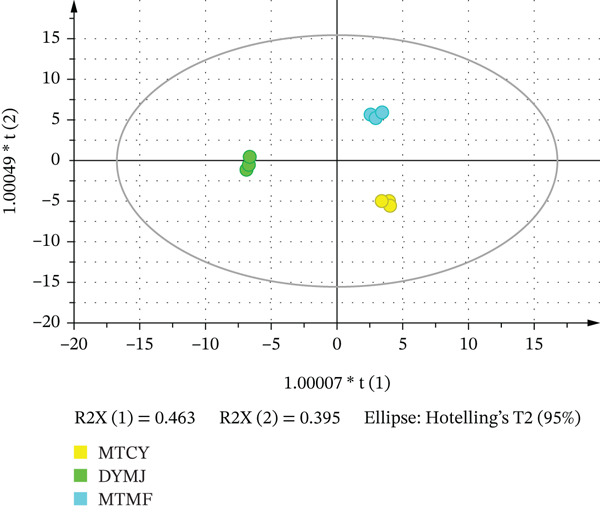
OPLS model results of the scatter plot.

To establish quantitative relationships between metabolite profiles and sensory characteristics, PLSR analysis was performed. The concentrations of 22 compounds with high rOAV values (rOAV ≥ 1) were used as predictor variables (*X*), while E‐tongue sensory parameters served as response variables (*Y*). The PLSR model demonstrated good predictive capability (Figure 5): *R* ^2^ *Y* (explained variance) = 0.89, *Q* ^2^ (predictive ability) = 0.82, and RMSEP (root mean square error of prediction) = 0.156. Permutation testing (200 iterations) confirmed model validity without overfitting (*Q* ^2^ intercept = −0.42, *R* ^2^ intercept = 0.15) (Figure 6). VIP analysis identified metabolites with VIP > 1 as significant contributors to sensory differentiation (Figure 4): Heptanal exhibited the highest VIP score (1.42), indicating its primary contribution to sensory variation. The standardized regression coefficient (*β* = 0.45) showed a positive association with green/grassy sensory perception. Butanol (VIP = 1.38, *β* = 0.38) and pentanol (VIP = 1.35, *β* = 0.35) were identified as candidate contributors to sweet and balsamic notes, respectively. 2‐Pentanol (VIP = 1.28, *β* = −0.28) showed a negative association with umami, suggesting its role in modulating savory perception. Methyl 3‐methylbutanoate (VIP = 1.22, *β* = 0.32) contributed to fruity characteristics, consistent with its known apple‐like aroma (Table 2). The Pearson correlation analysis revealed significant relationships between specific metabolites and sensory parameters (Figure 5 and Table 3). These correlations are derived from experimental data and provide quantitative evidence for metabolite–sensory relationships.

**Table 2:** VIP scores and regression coefficients for key metabolites in PLSR model (VIP > 1 indicates significant contribution to the model. *β* values indicate direction and magnitude of association).

Rank	Metabolite	VIP score	Regression coefficient (*β*)	Primary sensory association
1	Heptanal	1.42	0.45	Green/grassy
2	Butanol	1.38	0.38	Sweetness
3	Pentanol	1.35	0.35	Balsamic
4	2‐Pentanol	1.28	−0.28	Bitterness (negative)
5	Methyl 3‐methylbutanoate	1.22	0.32	Fruity
6	2‐Hexanone	1.18	0.25	Buttery
7	Benzaldehyde	1.15	0.22	Almond/nutty
8	6‐Methyl‐5‐hepten‐2‐one	1.08	0.18	Citrus

**Table 3:** Specific metabolites and sensory parameters.

Metabolite	Sensory parameter	*r*value	*p*value	95% confidence interval
Heptanal	Green perception	0.82	0.003	0.42–0.95
Butanol	Sweetness	0.75	0.012	0.28–0.93
2‐Pentanol	Bitterness	0.71	0.021	0.19–0.91
Pentanol	Astringency	0.68	0.031	0.14–0.90
Methyl 3‐methylbutanoate	Fruity perception	0.65	0.042	0.08–0.89

### 3.6. Analysis of Various Green Tea Samples Using E‐Tongue

**Figure 7:**
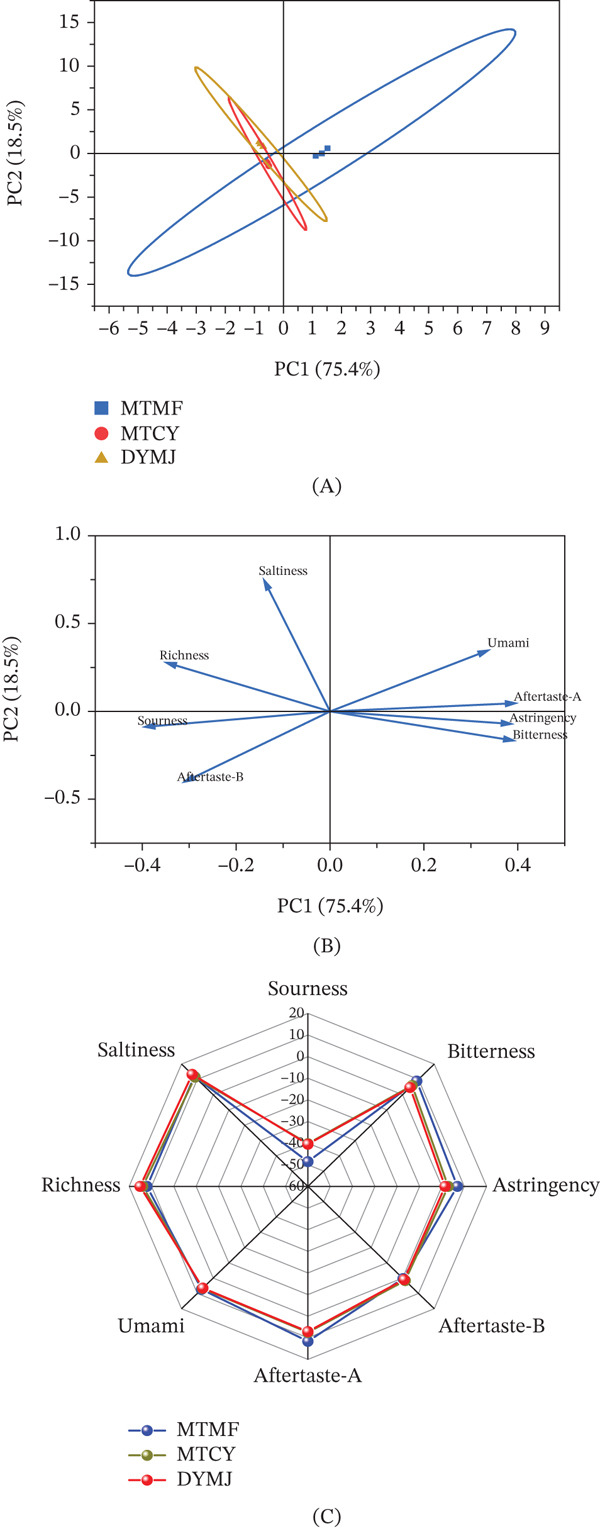
(A) E‐tongue PCA score plot, (B) horizontal PCA load plot, and (C) radar chart of the E‐tongue sensor responses to different types of tea.

The E‐tongue, which employs artificial lipid membrane technology, provides a quantitative assessment of aftertaste, encompassing key taste parameters such as acidity, bitterness, astringency, sweetness, saltiness, and umami [50]. A pivotal reference standard in this context is the tasteless node, representing the complete absence of taste, which was calibrated using a reference solution consisting of potassium chloride and tartaric acid. Although this solution exhibits slight acidity and salinity, it serves as a benchmark for comparative purposes. Taste values that fall below this tasteless threshold indicate an absence of perceptible taste in the sample [51]. As depicted in Figure 7, all analyzed samples exhibited statistically significant differences (*p* < 0.05) across attributes such as sourness, astringency, umami, bitterness, richness, aftertaste‐A, aftertaste‐B, and saltiness. Notably, the acidity levels for all three tea types were below −30, substantially below the perceptibility threshold for human taste. Conversely, the intensity of other taste attributes exceeded 0, placing them above the human taste detection threshold and serving as valid taste indicators. The aftertaste‐B measurements for all three tea types were marginally above the tasteless threshold, implying that this flavor attribute is minimal in the tea infusion and is unlikely to significantly affect the overall taste profile. Among the three tea types, MTMF demonstrated pronounced bitterness, astringency, and aftertaste‐A, with respective sensing values of 8.877 ± 0.196, 6.93 ± 1.679, and 11.63 ± 1.099 (Figure 7C). PCA was conducted on the response data from eight sensors for the three samples, as illustrated in Figure 7A. The substantial overlap observed among the three samples suggests negligible differences in their taste information. The explained variance ratios for PCA1 and PCA2 were 75.4% and 18.5%, respectively, yielding a cumulative variance contribution exceeding 80%. This indicates that these two principal components provide a robust representation of the sample’s taste characteristics. A loading plot (Figure 7B) identified three taste attributes making significant contributions to the sample’s overall taste profile: aftertaste‐A, astringency, and bitterness. In conclusion, electronic tongue analysis is effective in differentiating the taste characteristics of various green teas.

### 3.7. Analysis of Various Green Tea Samples Using UPLC‐Q‐TOF‐MSMS

Amino acids, comprising approximately 1%–4% of the dry weight of tea leaves, serve as pivotal indicators of green tea quality and significantly influence its aroma [52]. The analysis of three tea samples identified 23, 24, and 23 amino acids, respectively. The mass error (parts per million) is determined by comparing theoretical and detected values, with amino acids within a 5.0 ppm range considered effectively detected. The results show that 6 amino acids were identified in Tea 1 and Tea 3, while 11 were identified in Tea 2. Additionally, all identified amino acids were quantitatively analyzed using external standard methods, with detailed data presented in Tables 4, 5, and 6. Common amino acids found in all samples include theanine, proline, glutamic acid, lysine, and methionine. Notably, the content of glutamic acid in all samples was 99,600 *μ*g/kg, a finding closely related to the umami flavor in green tea [53–55], which is consistent with the results of the electronic tongue umami (7.31 ± 0.509) detection. Similarly, the content of theanine in all samples was 233,000 *μ*g/kg. Theanine is not only a key flavor component but also has cardiac muscle strengthening, diuretic, and vasodilatory effects and is used in the treatment of mental diseases and depression [56]. Specifically, aspartic acid (357,000 *μ*g/kg), L‐threonine (139,000 *μ*g/kg), and serine (92,500 *μ*g/kg) were uniquely detected in Tea 2. The taste characteristics of amino acids in tea vary, with some exhibiting sweetness and others bitterness, depending on their hydrophobicity. Amino acids with acidic side chains, such as aspartic acid, also contribute to the umami flavor [57]. L‐Threonine makes a significant contribution to the bitterness in tea, which is consistent with the prominent bitterness (8.877 ± 0.196) detected by the electronic tongue in Tea 2. High levels of serine are reported to provide astringency in tea, which is consistent with the prominent astringency detected by the electronic tongue in Tea 2 [58]. In summary, the application of UPLC‐Q‐TOF‐MSMS technology allows for effective differentiation of green tea samples based on their amino acid composition.

**Table 4:** MTMF list of amino acid types. “—” indicates “not detected” or “very low content.”

No.	Compounds	Formula	MW	m/z	PM	RT	Error (ppm)	MSMS	Concentration (*μ*g/kg)
1	o‐Phosphoserine	C_2_H_8_NO_4_P	141.067	186.0085	186.016	0.93	112.6	N/A	—
2	Taurine	C_5_H_9_NO_4_	147.134	126.0227	126.022	0.91	52.7	N/A	—
3	o‐Phosphoethanolamine	C_8_H_15_NO_4_	189.215	142.0267	142.026	0.89	3.8	N/A	—
4	Aspartic acid	C_7_H_14_N_2_O_3_	174.203	134.0452	134.045	1.06	92.8	N/A	—
5	Hydroxy‐L‐proline	C_5_H_9_NO_2_	115.134	132.0655	132.066	1.12	54.6	N/A	—
6	Threonine	C_5_H_11_NO_2_	117.15	120.0658	120.066	1.28	−0.1	N/A	—
7	Serine	C_6_H_14_N_2_O_2_	146.192	106.0499	106.05	0.9	51.7	N/A	—
8	Asparagine	C_4_H_8_N_2_O_3_	132.122	133.0611	133.061	0.92	22.6	N/A	—
9	Glutamic acid	C_5_H_9_NO_4_	147.134	148.0608	148.06	0.91	2.7	102.0552	99,600
10	Glutamine	C_10_H_18_N_2_O_5_	246.268	247.1336	247.129	2.31	19.2	N/A	—
11	Sarcosine	C_8_H_15_NO_4_	189.215	190.1081	190.107	0.89	3.8	158.0816	259,000
12	Theanine	C_7_H_14_N_2_O_3_	174.203	175.1082	175.108	1.06	2.8	N/A	233,000
13	Alpha‐aminoadipic acid	C_6_H_11_NO_4_	161.161	162.0766	162.076	0.96	3.3	N/A	—
14	Proline	C_5_H_9_NO_2_	115.134	116.0711	116.071	1.12	4.6	116.0709	186,000
15	Glycine	C_2_H_5_NO_2_	75.069	76.0479	76.039	5.19	112.6	N/A	—
16	Alanine	C_3_H_7_NO_2_	89.096	90.0549	90.055	0.95	−0.3	N/A	—
17	Citruline	C_6_H_13_N_3_O_3_	175.191	176.1115	176.103	1.03	48.7	N/A	—
18	a‐Amino‐n‐butyric acid	C_4_H_9_NO_2_	103.123	104.0707	104.071	0.95	20.8	104.1071	—
19	Valine	C_5_H_11_NO_2_	117.15	118.0862	118.086	1.28	−0.1	N/A	197,000
20	Cystine	C_6_H_12_N_2_O_4_S_2_	240.316	241.0373	241.031	0.95	25.7	N/A	—
21	Methionine	C_5_H_11_NO_2_S	149.22	N/A	150.058	1.72	N/A	N/A	—
22	Cystathionine	C_7_H_14_N_2_O_4_S	222.273	223.0646	223.075	1.99	−45.3	N/A	—
23	Tsoleucine	C_6_H_13_NO_2_	131.177	N/A	132.102	2.37	N/A	N/A	—
24	Leucine	C_6_H_13_NO_2_	131.177	N/A	132.102	2.37	N/A	N/A	—
25	Tyrosine	C_14_H_19_NO_5_	281.313	N/A	282.134	8.9	N/A	N/A	—
26	Phenylalanine	C_9_H_11_NO_2_	165.194	N/A	166.086	6.03	N/A	N/A	—
27	b‐Aminoisobutyric acid	C_4_H_9_NO_2_	103.123	104.0707	104.071	0.95	90.8	104.1071	—
28	Aminobutyric acid	C_4_H_9_NO_2_	103.123	104.0707	104.071	0.95	70.8	104.1071	—
29	Tryptophan 2	C_11_H_12_N_2_O_2_	204.231	N/A	205.097	2.01	N/A	N/A	—
30	Tryptophan	C_11_H_12_N_2_O_2_	204.231	N/A	205.097	2.01	N/A	N/A	—
31	Plusallo‐*σ*‐hydroxylysine	C_6_H_15_N_2_O_3_Cl	198.653	199.0774	199.084	7.77	−35.3	N/A	—
32	Ornithine	C_5_H_12_N_2_O_2_	132.165	133.1015	133.097	2.37	32.5	N/A	—
33	Lysine	C_6_H_14_N_2_O_2_	146.192	147.1131	147.113	0.9	1.7	N/A	183,000
34	1‐Methylhistidine	C_7_H_11_N_3_O_2_	169.186	170.0978	170.092	8.1	31.8	N/A	—
35	Histidine	C_6_H_9_N_3_O_2_	155.159	156.0660	156.077	1.07	−68.8	156.0658	—
36	3‐Methylhistidine	C_7_H_11_N_3_O_2_	169.186	170.0978	170.092	8.1	31.8	N/A	—
37	Anserine	C_10_H_16_N_4_O_3_	240.266	241.1359	241.13	2	26.3	196.0969	—
38	Carnosine	C_9_H_14_N_4_O_3_	226.239	227.1101	227.114	0.95	−16.5	N/A	—
39	Arginine	C_6_H_14_N_4_O_2_	174.206	175.1083	175.119	1	−61	158.0816	—
40	Isoleucine	C_6_H_13_NO_2_	131.177	N/A	132.102	2.37	N/A	N/A	—
41	Asparticum	C_4_H_7_NO_4_	133.107	134.0452	134.045	0.92	52.8	N/A	—

**Table 5:** MTCY list of amino acid types. “—” indicates “not detected” or “very low content.”

No.	Compounds	Formula	MW	m/z	PM	RT	Error (ppm)	MSMS	Concentration (*μ*g/kg)
1	o‐Phosphoserine	C_3_H_8_NO_6_P	185.078	186.0256	142.026	0.89	50.3	N/A	—
2	Taurine	C_2_H_7_NO_3_S	125.155	126.0212	134.045	1.13	−5.8	N/A	—
3	o‐Phosphoethanolamine	C_2_H_8_NO_4_P	141.067	142.0269	120.066	0.93	3.4	N/A	—
4	Aspartic acid	C_4_H_7_NO_4_	133.107	134.0459	106.05	1.54	1.3	N/A	357,000
5	Hydroxy‐L‐proline	C_5_H_9_NO_3_	131.134	N/A	133.061	2.07	N/A	N/A	—
6	Threonine	C_4_H_9_NO_3_	119.123	N/A	148.06	6.02	3.3	N/A	139,000
7	Serine	C_3_H_7_NO_3_	105.096	N/A	190.107	2.33	2.6	N/A	92,500
8	Asparagine	C_4_H_8_N_2_O_3_	132.122	133.0653	175.108	1.64	3.9	N/A	287,000
9	Glutamic acid	C_5_H_9_NO_4_	147.134	148.0608	162.076	0.93	2.8	102.0553	99,600
10	Glutamine	C_10_H_18_N_2_O_5_	246.268	N/A	116.071	2.31	N/A	N/A	—
11	Sarcosine	C_8_H_15_NO_4_	189.215	190.1080	147.113	0.91	3.4	N/A	259,000
12	Theanine	C_7_H_14_N_2_O_3_	174.203	175.1081	175.108	1.06	2.2	158.0811	233,000
13	Alpha‐aminoadipic acid	C_6_H_11_NO_4_	161.161	N/A	162.076	7.01	1.1	N/A	111,000
14	Proline	C_5_H_9_NO_2_	115.134	116.0708	116.071	1.12	1.8	116.0707	186,000
15	Glycine	C_2_H_5_NO_2_	75.069	76.0470	76.039	5.19	100.8	N/A	—
16	Alanine	C_3_H_7_NO_2_	89.096	90.0551	90.055	0.95	29.1	N/A	—
17	Citruline	C_6_H_13_N_3_O_3_	175.191	176.1116	176.103	1.36	49	N/A	—
18	a‐Amino‐n‐butyric acid	C_4_H_9_NO_2_	103.123	104.0707	104.071	0.95	5.6	N/A	—
19	Valine	C_5_H_11_NO_2_	117.15	118.0865	118.086	1.28	5.5	118.0866	—
20	Cystine	C_6_H_12_N_2_O_4_S_2_	240.316	N/A	241.031	0.95	N/A	N/A	—
21	Methionine	C_5_H_11_NO_2_S	149.22	150.0525	150.058	1.72	−38.6	N/A	—
22	Cystathionine	C_7_H_14_N_2_O_4_S	222.273	223.0645	223.075	1.99	−45.7	N/A	—
23	Tsoleucine	C_6_H_13_NO_2_	131.177	132.1105	132.102	2.37	65	N/A	—
24	Leucine	C_6_H_13_NO_2_	131.177	132.1105	132.102	2.37	65	N/A	—
25	Tyrosine	C_14_H_19_NO_5_	281.313	N/A	282.134	8.9	N/A	N/A	—
26	Phenylalanine	C_9_H_11_NO_2_	165.194	N/A	166.086	6.03	N/A	N/A	—
27	b‐Aminoisobutyric acid	C_4_H_9_NO_2_	103.123	104.0707	104.071	0.95	6.6	N/A	—
28	Aminobutyric acid	C_4_H_9_NO_2_	103.123	104.0707	104.071	0.95	7.6	N/A	—
29	Tryptophan 2	C_11_H_12_N_2_O_2_	204.231	205.0868	205.097	2.01	−50.4	N/A	—
30	Tryptophan	C_11_H_12_N_2_O_2_	204.231	205.0868	205.097	2.01	−50.4	N/A	—
31	Plusallo‐*σ*‐hydroxylysine	C_6_H_15_N_2_O_3_Cl	198.653	199.1705	199.084	7.77	432.6	N/A	—
32	Ornithine	C_5_H_12_N_2_O_2_	132.165	N/A	133.097	2.37	N/A	N/A	—
33	Lysine	C_6_H_14_N_2_O_2_	146.192	147.1131	147.113	0.9	2.0	130.0864	183,000
34	1‐Methylhistidine	C_7_H_11_N_3_O_2_	169.186	N/A	170.092	8.1	N/A	N/A	—
35	Histidine	C_6_H_9_N_3_O_2_	155.159	156.0752	156.077	6.52	−10.1	N/A	—
36	3‐Methylhistidine	C_7_H_11_N_3_O_2_	169.186	N/A	170.092	8.1	N/A	N/A	—
37	Anserine	C_10_H_16_N_4_O_3_	240.266	241.1553	241.13	2	107	196.0965	—
38	Carnosine	C_9_H_14_N_4_O_3_	226.239	227.1131	227.114	0.95	−3.4	N/A	—
39	Arginine	C_6_H_14_N_4_O_2_	174.206	175.1082	175.119	1	−61.4	158.0811	—
40	Isoleucine	C_6_H_13_NO_2_	131.177	132.1105	132.102	2.37	65	N/A	—
41	Asparticum	C_4_H_7_NO_4_	133.107	134.0459	134.045	1.54	8.3	N/A	—

**Table 6:** DYMJ list of amino acid types. “—” indicates “not detected” or “very low content.”

No.	Compounds	Formula	MW	m/z	PM	RT	Error (ppm)	MSMS	Concentration (*μ*g/kg)
1	o‐Phosphoserine	C_3_H_8_NO_6_P	185.078	186.0058	186.016	0.89	−56.0	N/A	—
2	Taurine	C_2_H_7_NO_3_S	125.155	126.0216	126.022	1.13	−2.6	N/A	—
3	o‐Phosphoethanolamine	C_2_H_8_NO_4_P	141.067	142.0278	142.026	0.93	9.8	N/A	—
4	Aspartic acid	C_4_H_7_NO_4_	133.107	N/A	134.045	1.54	N/A	N/A	—
5	Hydroxy‐L‐proline	C_5_H_9_NO_3_	131.134	N/A	132.066	2.07	N/A	N/A	—
6	Threonine	C_4_H_9_NO_3_	119.123	N/A	120.066	6.02	N/A	N/A	—
7	Serine	C_3_H_7_NO_3_	105.096	106.0569	106.05	2.33	66.5	N/A	—
8	Asparagine	C_4_H_8_N_2_O_3_	132.122	133.0648	133.061	1.64	30.1	N/A	—
9	Glutamic acid	C_5_H_9_NO_4_	147.134	148.0608	148.06	0.93	2.6	130.0498	99,600
10	Glutamine	C_10_H_18_N_2_O_5_	246.268	247.1330	247.129	2.31	16.9	N/A	—
11	Sarcosine	C_8_H_15_NO_4_	189.215	190.1080	190.107	0.91	3.2	190.0711	259,000
12	Theanine	C_7_H_14_N_2_O_3_	174.203	175.1081	175.108	1.06	2	158.0810	233,000
13	Alpha‐aminoadipic acid	C_6_H_11_NO_4_	161.161	N/A	162.076	7.01	N/A	N/A	—
14	Proline	C_5_H_9_NO_2_	115.134	116.0707	116.071	1.12	0.9	116.0711	186,000
15	Glycine	C_2_H_5_NO_2_	75.069	76.0469	76.039	5.19	99.8	N/A	—
16	Alanine	C_3_H_7_NO_2_	89.096	90.0550	90.055	0.95	6.5	N/A	—
17	Citruline	C_6_H_13_N_3_O_3_	175.191	176.1115	176.103	1.36	48.2	N/A	—
18	a‐Amino‐n‐butyric acid	C_4_H_9_NO_2_	103.123	104.0704	104.071	0.95	−2.4	N/A	—
19	Valine	C_5_H_11_NO_2_	117.15	118.0863	118.086	1.28	0.6	118.0863	197,000
20	Cystine	C_6_H_12_N_2_O_4_S_2_	240.316	N/A	241.031	0.95	N/A	N/A	—
21	Methionine	C_5_H_11_NO_2_S	149.22	150.0575	150.058	1.72	−5.8	N/A	—
22	Cystathionine	C_7_H_14_N_2_O_4_S	222.273	223.0644	223.075	1.99	−46.2	N/A	—
23	Tsoleucine	C_6_H_13_NO_2_	131.177	N/A	132.102	2.37	N/A	N/A	—
24	Leucine	C_6_H_13_NO_2_	131.177	N/A	132.102	2.37	N/A	N/A	—
25	Tyrosine	C_14_H_19_NO_5_	281.313	N/A	282.134	8.9	N/A	N/A	—
26	Phenylalanine	C_9_H_11_NO_2_	165.194	166.0934	166.086	6.03	43.3	N/A	—
27	b‐Aminoisobutyric acid	C_4_H_9_NO_2_	103.123	104.0704	104.071	0.95	−2.4	N/A	—
28	Aminobutyric acid	C_4_H_9_NO_2_	103.123	104.0704	104.071	0.95	−2.4	N/A	—
29	Tryptophan 2	C_11_H_12_N_2_O_2_	204.231	N/A	205.097	2.01	N/A	N/A	—
30	Tryptophan	C_11_H_12_N_2_O_2_	204.231	N/A	205.097	2.01	N/A	N/A	—
31	Plusallo‐*σ*‐hydroxylysine	C_6_H_15_N_2_O_3_Cl	198.653	199.1701	199.084	7.77	430.3	N/A	—
32	Ornithine	C_5_H_12_N_2_O_2_	132.165	133.1018	133.097	2.37	34.9	N/A	—
33	Lysine	C_6_H_14_N_2_O_2_	146.192	147.1131	147.113	0.9	2	130.0870	183,000
34	1‐Methylhistidine	C_7_H_11_N_3_O_2_	169.186	170.0977	170.092	8.1	31.3	N/A	—
35	Histidine	C_6_H_9_N_3_O_2_	155.159	156.0779	156.077	6.52	7.3	N/A	—
36	3‐Methylhistidine	C_7_H_11_N_3_O_2_	169.186	170.0977	170.092	8.1	31.3	N/A	—
37	Anserine	C_10_H_16_N_4_O_3_	240.266	N/A	241.13	2	N/A	N/A	—
38	Carnosine	C_9_H_14_N_4_O_3_	226.239	227.1085	227.114	0.95	−23.5	N/A	—
39	Arginine	C_6_H_14_N_4_O_2_	174.206	175.1081	175.119	1	−61.9	158.0810	—
40	Isoleucine	C_6_H_13_NO_2_	131.177	N/A	132.102	2.37	N/A	N/A	—
41	Asparticum	C_4_H_7_NO_4_	133.107	N/A	134.045	1.54	N/A	N/A	—

**Figure 8:**
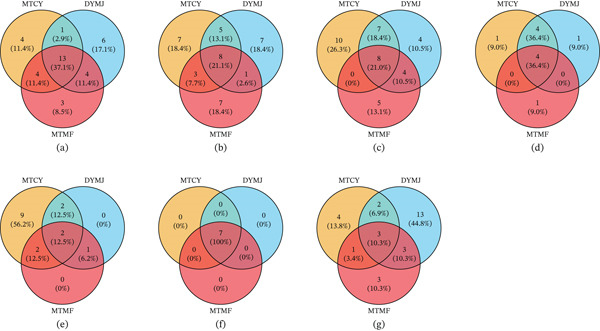
Venn diagram of the distribution of screened phenolic compounds. (a) Phenolic acids in MTCY, DYMJ, and MTMF. (b) Anthocyanin in MTCY, DYMJ, and MTMF. (c) Flacone in MTCY, DYMJ, and MTMF. (d) Flavonol in MTCY, DYMJ, and MTMF. (e) Flavonoid in MTCY, DYMJ, and MTMF. (f) Flavanol acids in MTCY, DYMJ, and MTMF. (g) Other polyphenols in MTCY, DYMJ, and MTMF.

UPLC‐Q‐TOF‐MSMS is a powerful tool that can detect both amino acids and polyphenolic substances in tea. Just like amino acids, polyphenolic substances make up about 30% of the dry weight of tea leaves [59]. These substances are known for their medicinal properties, including antioxidant effects, blood sugar regulation, and blood lipid reduction, which have been supported by both theoretical and clinical studies [60]. Tables 7, 8, and 9 delineate the spectrum of polyphenols identified within three distinct green tea samples. The identified compounds encompass phenolic acids, flavones, flavanols, flavonols, flavonoids, and anthocyanins, with additional compounds categorized under others. This latter category comprises complex and less frequently encountered substances, including benzophenones, lignans, and tannins. Using this advanced technology, researchers identified 159 polyphenolic compounds in total, with MTCY containing 115, DYMJ having 117, and MTMF consisting of 91. Interestingly, all three samples had 35 phenolic acid compounds in common, as depicted in Figure 8. The types of phenolic acids found in the samples were quite similar, with most being shared among MTCY, DYMJ, and MTMF (Figure 8a). Additionally, all three samples shared the same seven flavanol compounds, including L‐epicatechin, epiafzelechin, procyanidin trimer A‐type, procyanidin trimer, catechin, epicatechin, and dihydroquercetin taxifolin, which are known for their antioxidant properties and various health benefits [61]. As for flavonoids, MTCY had 16 detected, while DYMJ and MTMF had five in common. These compounds, similar to flavanols, have beneficial physiological activities and also exhibit anti‐inflammatory effects and the ability to regulate blood sugar and certain enzyme functions [62]. Just like in amino acid detection, UPLC‐Q‐TOF‐MSMS is indicated to be an effective method for detecting polyphenolic compounds and distinguishing between different green tea samples.

**Table 7:** Summary table of MTCY polyphenolic compound types.

Type	No.	Compounds	Formula	MW	m/z	PM	RT	Error (ppm)	MSMS
Phenolic acid	1	Gallic acid	C_7_H_6_O_5_	170.125	169.0152	169.014	1.36	5.5	90.9605
	2	Gallic acid‐6 hydroxy diphenyl‐pyran glucose	C_27_H_22_O_18_	634.473	633.0729	633.073	1.32	−0.6	633.0737
	3	Gallocatechin	C_15_H_14_O_7_	306.277	305.0679	305.067	1.41	4.2	90.9605
	4	Protocatechuic acid	C_7_H_6_O_4_	154.125	153.0198	153.019	1.32	3.2	N/A
	5	2‐5‐Dihydroxybenzoic acid	C_7_H_6_O_4_	154.125	153.0198	153.019	1.32	3.2	N/A
	6	p‐Hydroxybenzoic acid	C_7_H_6_O_3_	138.125	137.0257	137.024	1.4	9.6	N/A
	7	Chlorogenic acid	C_16_H_18_O_9_	354.320	353.0887	353.088	1.27	2.5	135.0453
	8	Vanillic acid	C_8_H_8_O_4_	168.152	167.0347	167.035	1.22	−1.4	N/A
	9	Caffeic acid	C_9_H_8_O_4_	180.163	179.0359	179.035	1.18	5	N/A
	10	Quinic acid	C_7_H_12_O_6_	192.173	191.0571	191.056	1.27	5.2	191.0567
	11	Malic acid	C_4_H_6_O_5_	134.092	133.0150	133.014	1.27	5.3	N/A
	12	Salicylic acid	C_7_H_6_O_3_	138.125	137.0257	137.024	1.4	9.6	N/A
	13	trans‐Cinnamic acid	C_9_H_8_O_2_	148.163	147.0457	147.045	1.22	3.8	N/A
	14	Myricetin‐rhamnose malic acid	C_25_H_24_O_16_	580.467	579.1001	577.12	1.3	27.6	N/A
	15	Quercetin‐hexose malic acid	C_25_H_24_O_16_	580.467	579.1001	579.099	1.3	1.7	N/A
	16	Genistein‐hexose malic acid	C_25_H_24_O_14_	548.467	547.1127	579.099	1.18	1.7	N/A
	17	Feruloyl quinic acid	C_17_H_20_O_9_	368.347	367.1034	367.103	1.19	0	N/A
	18	Caffeoylquinic acid	C_17_H_20_O_8_	352.347	353.0887	351.109	1.27	28.4	135.0453
	19	Chlorogenic acid	C_16_H_18_O_9_	354.32	353.0887	353.088	1.27	2.5	135.0453
	20	Coumaroyl quinic acid	C_16_H_18_O_8_	338.32	337.0941	337.093	1.31	3.6	337.2139
	21	Benzoic acid	C_7_H_6_O_2_	122.125	121.0297	121.03	1.27	1.5	N/A
	22	Quinic acid	C_7_H_12_O_6_	192.173	191.0571	191.056	1.27	5.2	191.0567
	23	5‐Caffeoylquinic acid	C_16_H_18_O_9_	354.32	353.0887	353.088	1.27	2.5	135.0453
	24	Hydroxyferulic acid	C_10_H_10_O_5_	210.19	209.0460	209.046	1.3	2.4	137.0238

Flavanol	25	L‐Epicatechin	C_15_H_14_O_6_	290.277	289.0729	289.072	1.36	3.8	203.0722
	26	Epiafzelechin	C_15_H_14_O_5_	274.277	273.0768	273.077	1.54	−0.2	N/A
	27	Procyanidin trimer A‐type	C_45_H_36_O_18_	864.783	863.1804	863.183	1.14	−2.8	N/A
	28	Procyanidin trimer	C_45_H_36_O_18_	864.783	863.1804	863.183	1.14	−2.8	N/A
	29	Catechin	C_15_H_14_O_6_	290.277	289.0729	289.072	1.36	3.8	303.0511
	30	Epicatechin	C_15_H_14_O_6_	290.277	289.0729	289.072	1.36	3.8	203.0722
	31	Dihydroquercetin taxifolin	C_15_H_12_O_7_	304.261	303.0518	303.051	1.39	2.6	N/A

Flacone	32	Quercetin‐methylpentoside‐dihexoside	C_33_H_40_O_21_	772.683	771.1996	771.199	1.34	0.9	N/A
	33	Quercetin‐O‐coumaroyl hexose	C_30_H_26_O_14_	610.538	609.1224	609.125	1.34	−4.3	N/A
	34	Kaempferol‐O‐coumaroyl hexose 1	C_30_H_26_O_13_	594.538	593.1301	593.13	1.17	0.1	N/A
	35	Quercetin‐3‐O‐b‐D‐xyloxyl‐a‐L‐rhamnoside	C_26_H_28_O_15_	580.51	579.1407	579.136	1.37	8.9	N/A
	36	Kaempferol‐O‐pentosylhexoside	C_26_H_28_O_15_	580.51	579.1407	579.136	1.37	8.9	N/A
	37	7‐O‐Methyl‐cyanidin‐3‐O‐galactoside	C_22_H_22_O_11_	462.418	461.1100	613.12	1.24	14.1	415.1596
	38	Epicatechin gallate	C_22_H_18_O_10_	442.386	441.0836	441.083	1.45	2	303.0511
	39	Epicatechin‐hexose	C_21_H_24_O_11_	452.423	451.1252	451.125	1.22	1.3	N/A
	40	Dihydroquercetin‐3‐glucoside	C_21_H_22_O_12_	466.407	465.1027	465.104	32.5	−2.4	N/A
	41	Apigenin glucoside	C_21_H_22_O_10_	434.407	433.1152	433.114	1.26	2.6	N/A
	42	Quercetin glucoside	C_21_H_20_O_12_	464.391	463.0884	463.088	1.34	0.4	N/A
	43	Kaempferol hexoside	C_21_H_20_O_12_	464.391	463.0884	463.088	1.34	0.4	N/A
	44	Delphinidin hexoside	C_21_H_20_O_12_	464.391	463.0884	463.088	1.34	0.4	N/A
	45	Quercetin‐3‐O‐b‐D‐glucoside	C_21_H_20_O_12_	464.391	463.0884	463.088	1.34	0.4	N/A
	46	Quercetin glucoside	C_21_H_20_O_12_	464.391	463.0884	463.088	1.34	0.4	N/A
	47	Cyanidin hexoside	C_21_H_20_O_11_	448.391	447.0932	447.093	1.19	−0.2	N/A
	48	Cyanidin 3‐galactoside	C_21_H_20_O_11_	448.391	447.0932	447.093	1.19	−0.2	N/A
	49	Petunidin 3‐arabinoside	C_21_H_20_O_11_	448.391	447.0932	447.093	1.19	−0.2	N/A
	50	Kaempferol‐3‐O‐b‐D‐glucoside	C_21_H_20_O_11_	448.391	447.0932	447.093	1.19	−0.2	N/A
	51	Kaempferol 3‐glucuronide	C_21_H_18_O_13_	478.375	447.0932	477.067	1.43	−7.1	N/A
	52	Dihydromyicetin	C_15_H_12_O_8_	320.261	319.0468	319.046	1.35	2.6	N/A
	53	Luteolin‐7‐O‐beta‐glucuronide	C_21_H_18_O_12_	462.375	461.0728	461.073	1.43	0.6	N/A
	54	Kaempferol‐3‐O‐glucuronide	C_21_H_18_O_12_	462.375	461.0728	461.073	1.43	0.6	N/A
	55	Chrysoeriol C‐hexoside‐C‐pentoside	C_27_H_30_O_15_	594.537	593.1508	593.151	1.24	−0.6	N/A
	56	Mangiferitin	C_13_H_8_O_6_	260.207	259.0238	259.025	1.35	−4	N/A
	57	Spinochrome A	C_12_H_8_O_7_	264.196	263.0219	263.02	1.46	8.2	N/A

Flavonol	58	Catechin	C_15_H_14_O_6_	290.277	289.0729	289.072	1.36	3.8	203.0722
	59	Myricitrin O‐gallate	C_28_H_24_O_16_	616.5	615.0996	615.099	1.45	0.8	N/A
	60	Myricetin	C_15_H_10_O_8_	318.245	317.0323	317.03	1.22	6.5	N/A
	61	Quercetin	C_15_H_10_O_7_	302.245	301.0370	301.035	32.48	5.3	N/A
	62	Quercetin‐3‐O‐rhamnoside	C_21_H_20_O_11_	448.391	447.0932	447.093	1.19	−0.2	N/A
	63	Kaempferol	C_15_H_10_O_6_	286.245	285.0417	285.04	1.32	4.2	N/A
	64	Viscidulin	C_15_H_10_O_7_	302.245	301.0370	301.035	32.48	5.3	N/A
	65	Myricetrin	C_15_H_10_O_8_	318.245	317.0323	317.03	1.22	6.5	N/A
	66	Quercetin diglucoside	C_27_H_30_O_17_	626.537	625.1407	625.141	1.21	−0.5	N/A

Flavonoids	67	Camellianin A	C_29_H_32_O_15_	620.575	619.1715	619.167	1.3	7.5	N/A
	68	Rutin	C_27_H_30_O_16_	610.537	609.1464	609.146	1.34	0.4	N/A
	69	Hyperoside	C_21_H_20_O_12_	464.391	463.0884	463.088	1.34	0.4	N/A
	70	Kaempferol rutinoside	C_27_H_30_O_15_	594.537	593.1508	593.151	1.24	−0.6	N/A
	71	Kaempferol‐3‐rutinoside	C_27_H_30_O_15_	594.537	593.1508	593.151	1.24	−0.6	N/A
	72	Kaempferol rutinoside	C_27_H_30_O_15_	594.537	593.1508	593.151	1.24	−0.6	N/A
	73	Myricetin‐3‐O‐rhamnoside	C_21_H_20_O_12_	464.391	463.0884	463.088	1.34	0.4	N/A
	74	Quercetin‐3‐O‐b‐D‐galactoside	C_21_H_20_O_12_	464.391	463.0884	463.088	1.34	0.4	N/A
	75	Apigenin glucuronide	C_21_H_20_O_11_	448.391	447.0932	447.093	1.19	−0.2	N/A
	76	Luteolin hexoside	C_21_H_20_O_11_	448.391	447.0932	447.093	1.19	−0.2	N/A
	77	Kaempferol‐3‐O‐b‐D‐ galactoside	C_21_H_20_O_11_	448.391	447.0932	447.093	1.19	−0.2	N/A
	78	Quercetin‐3‐O‐a‐L‐rhamnoside	C_21_H_20_O_11_	448.391	447.0932	447.093	1.19	−0.2	N/A
	79	Kaempferol hexoside or luteolin hexoside	C_21_H_20_O_11_	448.391	447.0932	447.093	1.19	−0.2	N/A
	80	Quercetin rhamnoside	C_21_H_20_O_11_	448.391	447.0932	447.093	1.19	−0.2	N/A
	81	Kaempferol‐3‐glucoside	C_21_H_20_O_10_	432.391	461.0728	447.093	1.19	−0.2	N/A
	82	Quercetin arabinoside	C_20_H_18_O_11_	434.364	433.0772	433.078	1.13	−1	N/A

Anthocyanin	83	Delphinidin 3‐rutinoside‐5‐glucoside	C_33_H_40_O_21_	772.683	771.1996	771.199	1.34	0.9	N/A
	84	Cyanidin 3‐O‐diglucoside‐glucosylrutinoside	C_33_H_40_O_20_	756.683	755.2046	755.204	1.19	0.8	N/A
	85	Procyanidin B4	C_30_H_26_O_12_	578.538	577.1358	577.135	1.17	1.2	577.1347
	86	Procyanidin B1	C_30_H_26_O_12_	578.538	577.1358	577.135	1.17	1.2	577.1347
	87	Procyanidin B2	C_30_H_26_O_12_	578.538	577.1358	577.135	1.17	1.2	577.1347
	88	Procyanidin B	C_30_H_26_O_12_	578.538	577.1358	577.135	1.17	1.2	577.1347
	89	Delphinidin 3‐5‐diglucoside	C_27_H_30_O_17_	626.537	625.1407	625.141	1.21	−0.5	N/A
	90	Delphinidin rhamnosylhexoside	C_27_H_30_O_16_	610.537	609.1464	609.146	1.34	0.4	N/A
	91	Cyanidin hexosyl hexoside	C_27_H_30_O_16_	610.537	609.1464	609.146	1.34	0.4	N/A
	92	Cyanidin 3‐sophoroside	C_27_H_30_O_16_	610.537	609.1464	609.146	1.34	0.4	N/A
	93	Delphinidin 3‐rutinoside	C_27_H_30_O_16_	610.537	609.1464	609.146	1.34	0.4	N/A
	94	Cyanidin 3‐laminaribioside	C_27_H_30_O_16_	610.537	609.1464	609.146	1.34	0.4	N/A
	95	Cyanidin 3‐5‐diglucoside	C_27_H_30_O_16_	610.537	609.1464	609.146	1.34	0.4	N/A
	96	Cyanidin rhamnosyl hexoside	C_27_H_30_O_15_	594.537	593.1508	593.151	1.24	−0.6	N/A
	97	Cyanidin 3‐glucosyl‐rhamnoside	C_27_H_30_O_15_	594.537	593.1508	593.151	1.24	−0.6	N/A
	98	Cyanidin 3‐rutinoside	C_27_H_30_O_15_	594.537	593.1508	593.151	1.24	−0.6	N/A
	99	Delphinidin acetylhexoside	C_23_H_22_O_13_	506.429	505.1008	505.099	1.3	4	N/A
	100	Isorhamnetin hexoside	C_22_H_22_O_12_	478.418	477.1061	477.104	1.13	4.7	N/A
	101	Galloylcyanidin‐glycoside	C_22_H_14_O_10_	438.354	437.0520	437.051	1.37	1.3	N/A
	102	Delphinidin 3‐galactoside	C_21_H_20_O_12_	464.391	463.0884	463.088	1.34	0.4	N/A
	103	Cyanidin 3‐glucoside	C_21_H_20_O_11_	448.391	447.0932	447.093	1.19	−0.2	N/A
	104	Delphinidin	C_15_H_10_O_7_	302.245	301.0370	301.035	32.48	5.3	N/A
	105	Cyanidin	C_15_H_10_O_6_	286.245	285.0417	285.04	1.32	4.2	N/A

Others	106	Epicatechin gallate	C_22_H_18_O_10_	442.386	441.0836	441.083	1.45	2	303.0511
	107	7‐Hydroxycoumarin	C_9_H_6_O_3_	162.147	161.0251	161.024	1.22	4.3	N/A
	108	Methyl gallate	C_8_H_8_O_5_	184.152	183.0301	183.03	1.39	1	N/A
	109	Pentagalloyl‐hexoside 3	C_41_H_32_O_26_	940.707	939.1125	939.111	1.32	1.7	N/A
	110	Tri‐galloyl‐hexoside	C_27_H_24_O_18_	636.489	635.0885	635.089	1.32	−0.8	465.0669
	111	Digalloyl‐hexoside	C_20_H_20_O_14_	484.38	483.0790	483.078	1.49	2	N/A
	112	O‐Galloylnorbergenin	C_20_H_18_O_13_	466.364	465.0690	465.067	1.5	3.2	N/A
	113	O‐Galloyl arbutin	C_19_H_20_O_11_	424.369	423.0950	423.093	1.35	3.9	N/A
	114	Wistin	C_23_H_24_O_10_	460.445	459.1322	459.13	1.23	5.6	N/A
	115	Pinoresinol	C_20_H_22_O_6_	358.396	357.1349	357.134	1.26	1.6	N/A

**Table 8:** Summary table of DYMJ polyphenolic compound types.

Type	No.	Compounds	Formula	MW	m/z	PM	RT	Error (ppm)	MSMS
Phenolic acid	1	Gallic acid	C_7_H_6_O_5_	170.125	169.0154	169.014	1.36	6.5	169.0148
	2	Gallic acid‐6 hydroxy diphenyl‐pyran glucose	C_27_H_22_O_18_	634.473	633.0743	633.073	1.32	1.4	633.0726
	3	Gallocatechin	C_15_H_14_O_7_	306.277	305.0680	305.067	1.41	4.3	96.9604
	4	Protocatechuic acid	C_7_H_6_O_4_	154.125	153.0198	153.019	1.32	2.9	N/A
	5	2‐5‐Dihydroxybenzoic acid	C_7_H_6_O_4_	154.125	153.0198	153.019	1.32	2.9	N/A
	6	Chlorogenic acid	C_16_H_18_O_9_	354.32	353.0892	353.088	1.27	3.8	N/A
	7	Vanillic acid	C_8_H_8_O_4_	168.152	167.0355	167.035	1.22	3	N/A
	8	Caffeic acid	C_9_H_8_O_4_	180.163	179.0364	179.035	1.18	8.1	N/A
	9	Syringic acid	C_9_H_10_O_5_	198.179	197.0465	197.046	1.26	4.9	N/A
	10	Quinic acid	C_7_H_12_O_6_	192.173	191.0572	191.056	1.27	5.9	191.0564
	11	Malic acid	C_4_H_6_O_5_	134.092	133.0153	133.014	1.27	7.6	N/A
	12	Malic acid	C_4_H_6_O_5_	134.092	133.0153	133.014	1.27	7.6	N/A
	13	Ellagic acid	C_14_H_6_O_8_	302.202	301.0004	300.999	32.42	4.8	N/A
	14	Digallic acid	C_14_H_10_O_9_	322.234	321.0271	321.025	1.32	6	N/A
	15	O‐Acetylsyringic acid	C_11_H_12_O_6_	240.217	239.0571	239.056	1.27	4.2	N/A
	16	Sinapic acid	C_11_H_12_O_5_	224.217	223.0623	223.061	1.28	5	N/A
	17	p‐Coumaric acid	C_9_H_8_O_3_	164.163	163.0408	163.04	1.31	4.2	N/A
	18	Diferulic acids	C_20_H_18_O_8_	386.364	385.0937	385.093	1.28	2.2	N/A
	19	Hydroxyferulic acid	C_10_H_10_O_5_	210.19	209.0464	209.046	1.3	4	N/A
	20	1‐3‐Dicaffeoylquinic acid	C_25_H_24_O_12_	516.467	515.1186	515.119	1.37	−1.7	N/A
	21	1‐5‐Dicaffeoylquinic acid	C_25_H_24_O_12_	516.467	515.1186	515.119	1.37	−1.7	N/A
	22	Methyl coumaroyl quinic acid	C_17_H_20_O_8_	352.347	351.1108	351.109	1.22	6.5	N/A
	23	Caffeoylquinic acid	C_16_H_18_O_9_	354.32	353.0892	353.088	1.27	3.8	N/A
	24	Chlorogenic acid	C_16_H_18_O_9_	354.32	353.0892	353.088	1.27	3.8	N/A
	25	Coumaroyl quinic acid	C_16_H_18_O_8_	338.32	337.0940	337.093	1.31	3.3	337.1086
	26	Benzoic acid	C_7_H_6_O_2_	122.125	121.0305	121.03	1.27	8.2	N/A
	27	Quinic acid	C_7_H_12_O_6_	192.173	191.0572	191.056	1.27	5.9	191.0564
	28	5‐Caffeoylquinic acid	C_16_H_18_O_9_	354.32	353.0892	353.088	1.27	3.8	N/A

Flacone	29	Quercetin‐methylpentoside‐dihexoside	C_33_H_40_O_21_	772.683	771.2003	771.199	1.34	1.7	772.2027
	30	Quercetin‐O‐coumaroyl hexose	C_30_H_26_O_14_	610.538	609.1262	609.125	1.34	2.1	423.0737
	31	Kaempferol‐O‐coumaroyl hexose	C_30_H_26_O_13_	594.538	593.1311	593.13	1.17	1.7	N/A
	32	Dihydroquercetin‐7‐rutinoside	C_27_H_32_O_16_	612.553	611.1571	611.162	1.26	−7.7	N/A
	33	Apigenin	C_27_H_30_O_14_	578.537	577.1562	577.156	1.37	−0.2	N/A
	34	7‐O‐Methyl‐cyanidin‐3‐O‐galactoside	C_22_H_22_O_11_	462.418	461.1087	461.109	1.24	−0.6	N/A
	35	Epicatechin gallate	C_22_H_18_O_10_	442.386	441.0841	441.083	1.45	3	289.0719
	36	Epicatechin‐hexose	C_21_H_24_O_11_	452.423	451.1239	451.125	1.22	−1.4	N/A
	37	Apigenin glucoside	C_21_H_22_O_10_	434.407	433.1144	433.114	1.26	0.8	N/A
	38	Quercetin glucoside	C_21_H_20_O_12_	464.391	463.0889	463.088	1.34	1.6	300.0293
	39	Kaempferol hexoside	C_21_H_20_O_12_	464.391	463.0889	463.088	1.34	1.6	300.0293
	40	Delphinidin hexoside	C_21_H_20_O_12_	464.391	463.0889	463.088	1.34	1.6	300.0293
	41	Delphinidin hexoside	C_21_H_20_O_12_	464.391	463.0889	463.088	1.34	1.6	300.0293
	42	Quercetin glucoside	C_21_H_20_O_12_	464.391	463.0889	463.088	1.34	1.6	300.0293
	43	Quercetin glucoside	C_21_H_20_O_12_	464.391	463.0889	463.088	1.34	1.6	300.0293
	44	Pelargonidin 3‐glucoside	C_21_H_20_O_10_	432.391	431.1001	431.098	1.27	3.9	N/A
	45	Apigenin glucoside I	C_21_H_20_O_10_	432.391	431.1001	431.098	1.27	3.9	N/A
	46	Kaempherol rhamnoside	C_21_H_20_O_10_	432.391	431.1001	431.098	1.27	3.9	N/A
	47	Quercetin‐O‐glucuronide	C_21_H_18_O_13_	478.375	477.0672	477.067	1.13	−0.6	N/A
	48	Quercetin glucuronide	C_21_H_18_O_13_	478.375	477.0672	477.067	1.13	−0.6	N/A
	49	Kaempferol 3‐glucuronide	C_21_H_18_O_12_	462.375	461.0733	461.073	1.43	1.6	N/A
	50	Dihydromyicetin	C_15_H_12_O_8_	320.261	483.0813	319.046	1.35	4.2	N/A
	51	Luteolin‐7‐O‐beta‐glucuronide	C_21_H_18_O_12_	462.375	461.0733	461.073	1.43	1.6	N/A
	52	Kaempferol‐3‐O‐glucuronide	C_21_H_18_O_12_	462.375	461.0733	461.073	1.43	1.6	N/A
	53	Chrysoeriol C‐hexoside‐C‐pentoside	C_27_H_30_O_15_	594.537	593.1528	593.151	1.24	2.8	N/A
	54	Mangiferitin	C_13_H_8_O_6_	260.207	259.0239	259.025	1.35	−3.6	N/A
Flavanol	55	L‐Epicatechin	C_15_H_14_O_6_	290.277	289.0730	289.072	1.36	4.2	N/A
	56	Epiafzelechin	C_15_H_14_O_5_	274.277	273.0779	273.077	1.54	3.7	N/A
	57	Procyanidin trimer A‐type	C_45_H_36_O_18_	864.783	863.1834	863.183	1.14	0.5	N/A
	58	Procyanidin trimer	C_45_H_36_O_18_	864.783	863.1834	863.183	1.14	0.5	N/A
	59	Catechin	C_15_H_14_O_6_	290.277	289.0730	289.072	1.36	4.2	N/A
	60	Epicatechin	C_15_H_14_O_6_	290.277	289.0730	289.072	1.36	4.2	N/A
	61	Dihydroquercetin taxifolin	C_15_H_12_O_7_	304.261	303.0527	303.051	1.39	5.6	N/A

Flavonol	62	Catechin	C_15_H_14_O_6_	290.277	289.0730	289.072	1.36	4.2	N/A
	63	Myricitrin O‐gallate	C_28_H_24_O_16_	616.5	615.1013	615.099	1.45	3.5	N/A
	64	Myricetin	C_15_H_10_O_8_	318.245	317.0312	317.03	1.22	2.9	N/A
	65	Quercetin	C_15_H_10_O_7_	302.245	301.0379	301.035	32.48	8.5	N/A
	66	Kaempferol	C_15_H_10_O_6_	286.245	285.0414	285.04	1.32	3.4	N/A
	67	Catechin glucoside	C_21_H_23_O_11_	451.415	450.1143	450.117	1.22	−5.4	N/A
	68	Viscidulin	C_15_H_10_O_7_	302.245	301.0379	301.035	32.48	8.5	N/A
	69	Myricetrin	C_15_H_10_O_8_	318.245	317.0312	317.03	1.22	2.9	N/A
	70	Quercetin diglucoside	C_27_H_30_O_17_	626.537	625.1416	625.141	1.21	0.9	N/A

Flavonoids	71	Rutin	C_27_H_30_O_16_	610.537	609.1470	609.146	1.34	1.4	423.0737
	72	Hyperoside	C_21_H_20_O_12_	464.391	463.0889	463.088	1.34	1.6	300.0293
	73	Kaempferol rutinoside	C_27_H_30_O_15_	594.537	593.1528	593.151	1.24	2.8	N/A
	74	Kaempferol‐3‐rutinoside	C_27_H_30_O_15_	594.537	593.1528	593.151	1.24	2.8	N/A
	75	Quercetin‐3‐O‐b‐D‐glucoside	C_21_H_20_O_12_	464.391	463.0889	463.088	1.34	1.6	300.0293

Anthocyanin	76	Myricetin‐3‐O‐rhamnoside	C_21_H_20_O_12_	464.391	463.0889	463.088	1.34	1.6	300.0293
	77	Quercetin‐3‐O‐b‐D‐galactoside	C_21_H_20_O_12_	464.391	463.0889	463.088	1.34	1.6	300.0293
	78	Procyanidin B4	C_30_H_26_O_12_	578.538	577.1360	577.135	1.17	1.4	577.1345
	79	Procyanidin B1	C_30_H_26_O_12_	578.538	577.1360	577.135	1.17	1.4	577.1345
	80	Procyanidin B2	C_30_H_26_O_12_	578.538	577.1360	577.135	1.17	1.4	577.1345
	81	Procyanidin B3	C_30_H_26_O_12_	578.538	577.1360	577.135	1.17	1.4	577.1345
	82	Petunidin rhamnosylhexoside	C_28_H_32_O_16_	624.564	623.1645	623.162	1.41	4.4	N/A
	83	Delphinidin 3‐5‐diglucoside	C_27_H_30_O_17_	626.537	625.1416	625.141	1.21	0.9	N/A
	84	Delphinidin rhamnosylhexoside	C_27_H_30_O_16_	610.537	609.1470	609.146	1.34	1.4	N/A
	85	Cyanidin hexosylhexoside	C_27_H_30_O_16_	610.537	609.1470	609.146	1.34	1.4	423.0737
	86	Cyanidin 3‐sophoroside	C_27_H_30_O_16_	610.537	609.1470	609.146	1.34	1.4	423.0737
	87	Delphinidin 3‐rutinoside	C_27_H_30_O_16_	610.537	609.1470	609.146	1.34	1.4	423.0737
	88	Cyanidin 3‐laminaribioside	C_27_H_30_O_16_	610.537	609.1470	609.146	1.34	1.4	423.0737
	89	Cyanidin 3‐5‐diglucoside	C_27_H_30_O_16_	610.537	609.1470	609.146	1.34	1.4	423.0737
	90	Cyanidin rhamnosylhexoside	C_27_H_30_O_15_	594.537	593.1528	593.151	1.24	2.8	N/A
	91	Cyanidin 3‐glucosyl‐rhamnoside	C_27_H_30_O_15_	594.537	593.1528	593.151	1.24	2.8	N/A
	92	Cyanidin 3‐rutinoside	C_27_H_30_O_15_	594.537	593.1528	593.151	1.24	2.8	N/A
	93	Delphinidin 3‐galactoside	C_21_H_20_O_12_	464.391	463.0889	463.088	1.34	1.6	300.0293
	94	Pelargonidin hexoside	C_21_H_20_O_10_	432.391	431.1001	431.098	1.27	3.9	N/A
	95	Cyanidin	C_15_H_10_O_6_	286.245	285.0414	285.04	1.32	3.4	N/A
	96	Pelargonidin‐3‐O‐glucoside (callistephin)	C_21_H_21_O_10_	433.399	432.1048	432.106	1.22	−3.2	N/A

Others	97	Epicatechin gallate	C_22_H_18_O_10_	442.386	441.0841	441.083	1.45	3	289.0719
	98	Syringate	C_9_H_10_O_5_	198.179	197.0465	197.046	1.26	4.9	N/A
	99	7‐Hydroxycoumarin	C_9_H_6_O_3_	162.147	161.0255	161.024	1.22	6.8	N/A
	100	Dimer of cyanidin 3‐glucoside	C_42_H_40_O_22_	896.782	895.1939	895.194	1.22	0.1	N/A
	101	Dimer of cyanidin 3‐galactoside	C_42_H_40_O_22_	896.782	895.1939	895.194	1.22	0.1	N/A
	102	Pentagalloyl‐hexoside	C_41_H_32_O_26_	940.707	939.1199	939.111	1.32	9.6	N/A
	103	Delphinidin 3‐rutinoside‐5‐glucoside	C_33_H_40_O_21_	772.683	771.2003	771.199	1.34	1.7	772.2027
	104	Cyanidin 3‐O‐diglucoside‐glucosylrutinoside	C_33_H_40_O_20_	756.683	755.2052	755.204	1.19	1.6	756.2087
	105	trans‐Cinnamic acid	C_9_H_8_O_2_	148.163	147.0464	147.045	1.22	8.8	N/A
	106	Kaempferol rutinoside	C_27_H_30_O_15_	594.537	593.1528	593.151	1.24	2.8	N/A
	107	Pelargonidin rhamnosylhexoside	C_27_H_30_O_14_	578.537	577.1562	577.156	1.37	−0.2	N/A
	108	Pelargonidin 3‐rutinoside	C_27_H_30_O_14_	578.537	577.1562	577.156	1.37	−0.2	N/A
	109	Tri‐galloyl‐hexoside	C_27_H_24_O_18_	636.489	635.0891	635.089	1.32	0.2	N/A
	110	Isorhamnetin hexoside	C_22_H_22_O_12_	478.418	477.1048	477.104	1.13	1.9	N/A
	111	Digalloyl‐hexoside	C_20_H_20_O_14_	484.38	483.0813	483.078	1.49	6.8	483.0766
	112	Caffeoylglucose	C_15_H_18_O_9_	342.309	341.0892	341.088	1.28	4.1	341.1106
	113	Delphinidin	C_15_H_10_O_7_	302.245	301.0379	301.035	32.48	8.5	N/A
	114	1‐2‐O‐Dicaffeoylglycerol	C_25_H_24_O_12_	516.467	515.1186	515.119	1.37	−1.7	N/A
	115	Ajugol	C_15_H_24_O_9_	348.357	347.1360	347.135	1.19	3.7	N/A
	116	Pinoresinol	C_20_H_22_O_6_	358.396	357.1353	357.134	1.26	2.5	311.0970
	117	Galloylpyrogallol	C_13_H_10_O_7_	278.223	277.0381	277.035	1.28	9.8	N/A

**Table 9:** Summary table of MTMF polyphenolic compound types.

Type	No.	Compounds	Formula	MW	M\Z	RT	Error (ppm)	MSMS
Phenolic acid	1	Gallic acid	C_7_H_6_O_5_	170.125	169.0152	1.36	5.6	169.0138
	2	Gallocatechin	C_15_H_14_O_7_	306.277	305.0678	1.41	3.6	139.0406
	3	Protocatechuic acid	C_7_H_6_O_4_	154.125	153.0203	1.32	6.2	N/A
	4	2‐5‐Dihydroxybenzoic acid	C_7_H_6_O_4_	154.125	153.0203	1.32	6.2	N/A
	5	p‐Hydroxybenzoic acid	C_7_H_6_O_3_	138.125	137.0256	1.4	8.5	N/A
	6	Hydroxy methoxy dimethylbenzoic acid	C_10_H_12_O_4_	196.206	195.0674	1.26	5.9	N/A
	7	Chlorogenic acid	C_16_H_18_O_9_	354.32	353.0890	1.27	3.2	N/A
	8	Vanillic acid	C_8_H_8_O_4_	168.152	167.0354	1.22	2.2	N/A
	9	Caffeic acid	C_9_H_8_O_4_	180.163	179.0357	1.18	4.1	N/A
	10	Quinic acid	C_7_H_12_O_6_	192.173	191.0568	1.27	3.7	N/A
	11	Salicylic acid	C_7_H_6_O_3_	138.125	197.0553	1.4	8.5	N/A
	12	Malic acid	C_4_H_6_O_5_	134.092	133.0152	1.27	6.9	N/A
	13	Genistein‐hexose malic acid	C_25_H_24_O_14_	548.467	547.1125	1.18	5.9	N/A
	14	1‐3‐Dicaffeoylquinic acid	C_25_H_24_O_12_	516.467	515.1206	1.37	2.2	N/A
	15	1‐5‐Dicaffeoylquinic acid	C_25_H_24_O_12_	516.467	515.1206	1.37	2.2	N/A
	16	Caffeoylquinic acid	C_19_H_16_O_7_	356.337	353.0890	1.27	20.7	N/A
	17	Chlorogenic acid	C_16_H_18_O_9_	354.32	353.0890	1.27	3.2	N/A
	18	Coumaroyl quinic acid	C_16_H_18_O_8_	338.32	337.0929	1.31	0.1	337.0939
	19	Benzoic acid	C_7_H_6_O_2_	122.125	121.0305	1.27	8.3	N/A
	20	B2 Benzoic acid	C_30_H_24_O_13_	592.522	591.1181	1.37	6.2	N/A
	21	Quinic acid	C_7_H_12_O_6_	192.173	191.0568	1.27	3.7	191.0559
	22	5‐Caffeoylquinic acid	C_16_H_18_O_9_	354.32	353.0890	1.27	3.2	N/A
	23	Caffeoylmalic acid	C_13_H_12_O_8_	296.239	295.0471	1.14	3.8	N/A
	24	p‐Coumaric acid	C_9_H_8_O_3_	164.163	163.0408	1.31	4.2	N/A
	25	Hydroxyferulic acid	C_10_H_10_O_5_	210.19	209.0473	1.3	8.6	N/A
	26	Digallic acid	C_14_H_10_O_9_	322.234	385.0988	1.32	1.3	N/A

Flacone	27	Quercetin‐methylpentoside‐dihexoside	C_33_H_40_O_21_	772.683	771.1999	1.34	1.3	N/A
	28	Quercetin glucoside	C_21_H_20_O_12_	464.391	463.0889	1.34	1.6	N/A
	29	Kaempferol‐O‐coumaroyl hexose	C_30_H_26_O_13_	594.538	593.1309	1.17	1.5	N/A
	30	Apigenin	C_27_H_30_O_14_	578.537	577.1566	1.37	0.5	N/A
	31	Epicatechin gallate	C_22_H_18_O_10_	442.386	441.0837	1.45	2.2	290.0764
	32	Epicatechin‐hexose	C_21_H_24_O_11_	452.423	451.1254	1.22	1.8	N/A
	33	Apigenin glucoside	C_21_H_22_O_10_	434.407	433.1143	1.26	0.6	N/A
	34	Quercetin glucoside	C_21_H_20_O_12_	464.391	463.0889	1.34	1.6	N/A
	35	Kaempferol hexoside or luteolin hexoside	C_21_H_20_O_12_	464.391	463.0889	1.34	1.6	N/A
	36	Delphinidin hexoside	C_21_H_20_O_12_	464.391	463.0889	1.34	1.6	N/A
	37	Quercetin glucoside	C_21_H_20_O_12_	464.391	463.0889	1.34	1.6	N/A
	38	Quercetin‐O‐glucuronide	C_21_H_18_O_13_	478.375	477.0674	1.13	−0.1	N/A
	39	Quercetin glucuronide	C_21_H_18_O_13_	478.375	477.0674	1.13	−0.1	N/A
	40	O‐Galloylnorbergenin	C_20_H_18_O_13_	466.364	465.0698	1.5	4.9	N/A
	41	Quercetin arabinoside	C_20_H_18_O_11_	434.364	433.0797	1.13	4.8	N/A
	42	Dihydromyicetin	C_15_H_12_O_8_	320.261	319.0468	1.35	2.8	N/A
	43	Luteolin 7‐3‐disulfate	C_15_H_10_O_12_S_2_	446.385	444.9498	1.62	−9.8	N/A
	44	Quercetin diglucoside	C_27_H_30_O_17_	626.537	625.1415	1.21	0.8	N/A
	45	Mangiferitin	C_13_H_8_O_6_	260.207	259.0237	1.35	−4.5	N/A

Flavanol	46	L‐Epicatechin	C_15_H_14_O_6_	290.277	289.0729	1.36	4	245.0828
	47	Epiafzelechin	C_15_H_14_O_5_	274.277	273.0773	1.54	1.7	N/A
	48	Procyanidin trimer A‐type	C_45_H_36_O_18_	864.783	863.1833	1.14	0.5	N/A
	49	Procyanidin trimer	C_45_H_36_O_18_	864.783	863.1833	1.14	0.5	N/A
	50	Catechin	C_15_H_14_O_6_	290.277	289.0729	1.36	4	245.0828
	51	Epicatechin	C_15_H_14_O_6_	290.277	289.0729	1.36	4	245.0828
	52	Dihydroquercetin taxifolin	C_15_H_12_O_7_	304.261	303.0522	1.39	3.8	N/A

Flavonol	53	Catechin	C_15_H_14_O_6_	290.277	289.0729	1.36	4	245.0828
	54	Isorhamnetin hexoside	C_22_H_22_O_12_	478.418	477.1047	1.13	1.8	N/A
	55	Quercetin	C_15_H_10_O_7_	302.245	301.0363	32.48	3.2	N/A
	56	Kaempferol	C_15_H_10_O_6_	286.245	285.0414	1.32	3.1	N/A
	57	Viscidulin	C_15_H_10_O_7_	302.245	301.0363	32.48	3.2	N/A

Flavonoids	58	Rutin	C_27_H_30_O_16_	610.537	609.1459	1.34	−0.3	609.1444
	59	Hyperoside	C_21_H_20_O_12_	464.391	463.0889	1.34	1.6	N/A
	60	Quercetin‐3‐O‐b‐D‐glucoside	C_21_H_20_O_12_	464.391	463.0889	1.34	1.6	N/A
	61	Myricetin‐3‐O‐rhamnoside	C_21_H_20_O_12_	464.391	463.0889	1.34	1.6	N/A
	62	Quercetin‐3‐O‐b‐D‐galactoside	C_21_H_20_O_12_	464.391	463.0889	1.34	1.6	N/A

Anthocyanin	63	Delphinidin 3‐rutinoside‐5‐glucoside	C_33_H_40_O_21_	772.683	771.1999	1.34	1.3	N/A
	64	Cyanidin 3‐O‐diglucoside‐glucosylrutinoside	C_33_H_40_O_20_	756.683	755.2049	1.19	1.2	755.2030
	65	Procyanidin	C_30_H_26_O_12_	578.538	577.1367	1.17	2.7	N/A
	66	Proacyanidin dimer A	C_30_H_24_O_12_	576.522	575.1198	1.32	0.5	N/A
	67	7‐O‐Methyl‐delphinidin‐3‐O‐2‐galloyl‐galactoside	C_29_H_26_O_16_	630.527	629.1151	1.35	0.5	N/A
	68	7‐O‐Methyl‐cyanidin‐3‐O‐2‐galloyl‐galactoside	C_29_H_26_O_15_	614.527	613.1218	1.49	3.1	N/A
	69	Cyanidin galloylhexoside	C_28_H_24_O_15_	600.5	599.1047	1.37	0.8	N/A
	70	Cyanidin‐3‐O‐2‐galloyl‐galactoside	C_28_H_24_O_15_	600.5	599.1047	1.37	0.8	N/A
	71	Delphinidin 3‐5‐diglucoside	C_27_H_30_O_17_	626.537	625.1415	1.21	0.8	N/A
	72	Delphinidin rhamnosylhexoside	C_27_H_30_O_16_	610.537	609.1459	1.34	−0.3	609.1444
	73	Cyanidin hexosylhexoside	C_27_H_30_O_16_	610.537	609.1459	1.34	−0.3	609.1444
	74	Cyanidin 3‐sophoroside	C_27_H_30_O_16_	610.537	609.1459	1.34	−0.3	609.1444
	75	Delphinidin 3‐rutinoside	C_27_H_30_O_16_	610.537	609.1459	1.34	−0.3	609.1444
	76	Cyanidin 3‐laminaribioside	C_27_H_30_O_16_	610.537	609.1459	1.34	−0.3	609.1444
	77	Cyanidin 3‐5‐diglucoside	C_27_H_30_O_16_	610.537	609.1459	1.34	−0.3	609.1444
	78	Dihydroquercetin‐3‐glucoside	C_21_H_22_O_12_	466.407	465.1070	32.5	6.8	N/A
	79	Delphinidin 3‐galactoside	C_21_H_20_O_12_	464.391	463.0889	1.34	1.6	N/A
	80	Delphinidin	C_15_H_10_O_7_	302.245	301.0363	32.48	3.2	N/A
	81	Cyanidin	C_15_H_10_O_6_	286.245	285.0414	1.32	3.1	N/A

Others	82	7‐Hydroxycoumarin	C_9_H_6_O_3_	162.147	161.0253	1.22	5.5	N/A
	83	Methyl gallate	C_8_H_8_O_5_	184.152	183.0300	1.39	0.8	N/A
	84	Pentagalloyl‐hexoside	C_41_H_32_O_26_	940.707	939.1000	1.32	−11.7	N/A
	85	Pelargonidin rhamnosyl hexoside	C_27_H_30_O_14_	578.537	577.1566	1.37	0.5	N/A
	86	Pelargonidin 3‐rutinoside	C_27_H_30_O_14_	578.537	577.1566	1.37	0.5	N/A
	87	Tri‐galloyl‐hexoside	C_27_H_24_O_18_	636.489	635.0900	1.32	1.7	N/A
	88	Galloyl‐valoneic acid bilactone	C_22_H_22_O_21_	622.418	621.0544	1.31	−6	N/A
	89	1‐2‐O‐Dicaffeoylglycerol	C_25_H_24_O_12_	516.467	515.1206	1.37	2.2	N/A
	90	Levoglucosan gallate	C_13_H_14_O_9_	314.255	313.0574	1.21	3	N/A
	91	Epicatechin gallate	C_22_H_18_O_10_	442.386	441.0837	1.45	2.2	290.0764

The flavor characteristics of tea soup are inherently complex, arising not from a singular attribute but from the collective manifestation of multiple flavor compounds. Variations in the concentration of one compound can modulate the intensity of other flavor components, illustrating both synergistic and antagonistic interactions [63]. Catechins serve as the principal contributors to the bitterness and astringency profile of tea infusions [64], whereas theanine, glutamic acid, and aspartic acid are identified as key constituents associated with the umami taste [65]. Findings by Liu et al. suggest that umami‐active compounds can attenuate bitter sensations, and the interactions between catechins and glutamic acid, as well as between aspartic acid and glutamic acid, positively influence bitterness perception [66]. Furthermore, research conducted by Sun et al. revealed that low concentrations of monosodium glutamate augment the astringency induced by catechins, whereas elevated concentrations mitigate this effect [67]. Polyphenolic compounds are inherently colorless. However, during the tea processing stage, their exposure to oxygen, light, and elevated temperatures associated with oxidation results in the formation of colored compounds. This chemical transformation consequently alters both the visual appearance and sensory profile of the tea infusion [68]. Flavanols present in green tea, owing to their water solubility, readily dissolve into the brewed infusion, thereby constituting the principal source of its characteristic refreshing and stimulating attributes [69]. Anthocyanins are primarily associated with the astringent sensation, phenolic acids predominantly contribute to sourness, while flavonols mainly impart sweetness. The complex interplay among multiple constituents within the tea infusion ultimately yields a more nuanced and balanced taste perception [70, 71].

## 4. Discussion

This study demonstrates that the integration of metabolomics with sensory analysis can transform descriptive geographical comparison into quantitative, predictive research. Several methodological advancements distinguish our approach from previous tea metabolomics studies: (1) We established a PLSR model linking metabolite concentrations to electronic tongue sensory parameters. Unlike prior GC‐IMS studies, which primarily focused on compound identification and geographical discrimination [72, 73], our framework enables the prediction of sensory attributes directly from metabolite profiles. The model performance metrics (*R* ^2^ *Y* = 0.89, *Q* ^2^ = 0.82) indicate that key aroma compounds quantitatively explain a substantial proportion of sensory variance among the tested teas. (2) Our approach provides statistical evidence for metabolite–sensory associations derived from experimental data, rather than relying solely on literature inference. The significant correlations between specific metabolites and sensory parameters, calculated from our proprietary dataset, represent robust quantitative associations within our sample set. (3) VIP analysis ranked metabolites by their respective contributions to sensory variance, offering a quantitative basis for identifying the major aroma contributors.

Compared to previous GC‐IMS investigations on green teas [7, 20], our study extends far beyond mere compound identification. The identification of 22 compounds with high rOAV values (rOAV ≥ 1) facilitates a more targeted understanding of aroma‐active components, surpassing the broad compound lists reported in earlier studies. Furthermore, our comprehensive phenolic profiling (159 compounds) significantly expands the metabolite coverage compared to prior LC‐MS studies on similar tea varieties.

Regional amino acid profiling: Tables 10 and 11 provide a comparative analysis of amino acid profiles across regional green teas. Notably, Duyun Maojian Xia tea from Guizhou Province, renowned for its floral fruity nuances, exhibits a surprisingly limited amino acid composition, with only theanine being detected. Consequently, it displays a significantly lower amino acid diversity compared to the MTCY and MTMF green teas [74].

**Table 10:** This is a compilation of research on different varieties of green tea, including their respective origins, odors, volatile compounds, amino acids, and related references.

Green tea varieties	Origin	Amino acid	Volatiles	Taste	Reference
Duyun Maojian summer green tea	Duyun City, Guizhou Province	High content of amino acids, especially theanine	Alcohols, aldehydes, ketones, esters, alkenes, acids, heterocyclic compounds	Bitter, savory, sweet, floral fruity aroma	[74]
“Fuzao 2” tea	Japan	Sixteen amino acids were detected, including aspartic acid, glutamic acid, serine, threonine, glycine, alanine, cysteine, valine, methionine, isoleucine, leucine, tyrosine, phenylalanine, histidine, lysine, and arginine	—	Umami, bitter, astringent, sweet, floral, fruity, fragrant	[75]
Fuliang green tea	Fuliang area, Jiangxi Province	Sixteen amino acids were detected, including tryptophan, phenylalanine, lysine, threonine, isoleucine, leucine, valine, methionine, glutamic acid, aspartic acid, serine, histidine, arginine, tyrosine, alanine, and cysteine	Aldehydes, alcohols, ketones, esters, hydrocarbons, acids	Refreshing, fresh, bitter, and sweet, but with a certain degree of decay and burnt, floral, and fruity flavors, accompanied by dry wood, spicy, and burnt odors	[26]
Lu’an Guapian green tea	Jinzhai County, Anhui Province	—	139 volatile compounds, including aldehydes, alcohols, ketones, esters, heterocycles, terpenes, benzene, oxides, sulfides, and acids	Bitter, savory, bean aroma, floral aroma, fruity aroma	[4]
Qianlin green tea	Hubei Province Shenlong Frame	Thirteen amino acids were detected, excluding theanine, but containing alanine, arginine, asparagine, aspartic acid, glutamic acid, leucine, phosphatidylserine, serine, threonine, valine, gamma aminobutyric acid, 1‐methylhistidine, and isoleucine	40 volatile compounds, including alcohols, aldehydes, ketones, esters, terpenes, and heterocycles	Fresh, mellow, apple aroma	[12]
“Huangshanzhong” green tea	Qingdao City, Shandong Province	Detected 11 types of amino acids, including serine, lysine, proline, and so forth	Terpenes, heterocycles, esters, ketones, hydrocarbons, alcohols, aldehydes, hydrocarbons, amines, acids, phenols	Rich and savory, sweet, bitter, and bean flavored	[14]
Taiping Houkui green tea	Mount Huangshan, Anhui Province	25 types of amino acids, including theanine, aspartic acid, threonine, serine, glutamic acid, glutamine, glycine, alanine, alpha aminobutyric acid, cysteine, methionine, beta alanine, beta aminoisobutyric acid, proline, valine, isoleucine, leucine, tyrosine, phenylalanine, gamma aminobutyric acid, lysine, histidine, arginine, and citrulline	Alcohols, aldehydes, ketones, esters, aromatic compounds, hydrocarbons, terpenes	Fresh, mellow, floral, sweet, bitter	[76]; [77]; [78]
Longjing‐changye	Hangzhou City, Zhejiang Province	Tryptophan, methionine, serine, histidine, glutamine, arginine, and so forth	74 volatile substances, including aldehydes, ketones, alcohols, esters, hydrocarbons, aromatics, heterocyclic compounds, phenols, and acids	Fresh, bitter, astringent, floral, fruity flavors	[73]
Liupao tea	Liupao Town, Guangxi Province	—	165 volatile compounds, including terpenes, heterocycles, esters, ketones, hydrocarbons, alcohols, aromatics, acids, and amines	Sweet, floral, fruity, woody, aged, betel nut aroma	[72]
Big‐leaf green tea	Yichang City, Hubei Province	—	94 volatile compounds, including hydrocarbons, terpenes, aromatics, ketones, esters, alcohols, heterocyclic compounds, aldehydes, acids, amines, and nitrogen‐containing compounds	Sweet, floral, flavorful, fruity, mint, woody, astringent	[79]
MTMF	Meitan District, Guizhou Province	6 types of amino acids, o‐phosphoethanolamine, glutamic acid, sarcosine, theanine, proline, valine, lysine	25 volatile compounds, including alcohols, aldehydes, ketones, esters, hydrocarbons, and heterocycles	—	This study
MTCY	Meitan District, Guizhou Province	11 amino acids, o‐phosphoethanolamine, aspartic acid, threonine, serine, asparagine, glutamic acid, sarcosine, theanine, alpha‐aminoadipic acid, proline, lysine	59 volatile compounds, including alcohols, aldehydes, ketones, esters, hydrocarbons, and heterocycles	—	This study
DYMJ	Meitan District, Guizhou Province	6 types of amino acids, glutamic acid, sarcosine, theanine, proline, valine, lysine	38 volatile compounds, including dimethyl sulfide, methylallyl sulfide, 2‐ethyl‐3,5‐dimethylpyrazine, myrcene‐D, myrcene‐M, hexanenitrile, 4‐methyl‐2,5‐dimethyl‐3‐furanone, and 6‐methyl‐5‐hepten‐2‐one	—	This study

**Table 11:** Key novel contributions of this study.

Aspect	Previous studies	This study	Novel contribution
Analytical approach	GC‐IMS or LC‐MS alone [20]	Integrated HS‐GC‐IMS + UPLC‐Q‐TOF‐MSMS	Comprehensive volatile and nonvolatile profiling
VOC analysis	Descriptive compound lists [7]	rOAV calculation + PLSR sensory correlation	Quantitative metabolite–sensory prediction model
Statistical method	PCA/PLS‐DA for discrimination [74]	PLSR for prediction + VIP for contribution	Predictive capability with mechanistic insights
Sensory linkage	Literature‐based inference [12]	Data‐driven correlation (*r*, *p* values)	Statistical evidence from experimental data
Marker identification	General compound lists [76]	22 key compounds (rOAV ≥ 1) + unique markers	Targeted markers with quantitative ranking
Phenolic coverage	~50–100 compounds [77]	159 identified compounds	Most comprehensive profile for Guizhou teas
Practical application	Limited [78]	Validated classification model (*R* ^2^ *Y* = 0.89, *Q* ^2^ = 0.82)	Tool for quality assessment and authentication

Additional insights from Tables 10 and 11 reveal that Taiping Houkui green tea from Huangshan, Anhui Province, is characterized by its unique orchid fragrance, primarily attributed to alcohols, aldehydes, and ketones [76–78]. In contrast, Huangshan Zhong green tea from Qingdao, Shandong Province, possesses a distinct floral aroma dominated by heterocyclic, amine, and hydrocarbon compounds [14]. This flavor profile contrasts markedly with that of the Meitan green teas discussed earlier

## 5. Conclusions

This investigation elucidates metabolite profiles associated with aromatic variations among three Guizhou green tea cultivars and establishes a quantitative framework for metabolite–sensory correlation analysis. The principal findings demonstrate that (1) comprehensive analysis identified 57 VOCs and 159 phenolic compounds, with statistically significant differentiation in metabolite profiles among cultivars (OPLS‐DA: *R* ^2^ *Y* = 99.3*%*, *Q* ^2^ = 98.2*%*). (2) Compounds exhibiting high rOAV values (≥ 1) demonstrate predictive capability in explaining sensory variations through PLSR modeling (*R* ^2^ *Y* = 0.89, *Q* ^2^ = 0.82). (3) Specific metabolites exhibit significant correlations with sensory parameters: Heptanal correlates with green perception (*r* = 0.82, *p* = 0.003), butanol with sweetness (*r* = 0.75, *p* = 0.012), and 2‐pentanol with bitterness (*r* = 0.71, *p* = 0.021). (4) Distinctive metabolite combinations were identified for each tea cultivar, providing potential authentication markers. (5) Absolute quantification of 22 key aroma compounds and 15 major phenolics was achieved with comprehensive method validation. Future research should prioritize validation through sensory recombination and omission experiments to establish causal relationships, while expanded sample sets and integration with human sensory panels would enhance model generalizability and practical applicability.

**Nomenclature**
VOCs: volatile organic compounds
rOAVs: relative odor activity values
LOD: limits of detection
LOQ: limits of quantification
PLSR: partial least squares regression
RT: retention time
MF: molecular weight
PM: precursor mass
DT: drift time
OPLS‐DA: orthogonal partial least squares discriminant analysis
*R* ^2^ *Y*
: cumulative model variation in *Y*
*R* ^2^
: cumulative predicted variation
PLS‐DA: partial least squares discriminant analysis
VIP: variable importance in projection
HS‐GC‐IMS: Headspace–gas chromatography–ion mobility spectrometry
UPLC‐Q‐TOF‐MSMS: ultra‐performance liquid chromatography‐quadrupole time of flight tandem mass spectrometry

## Author Contributions

**Hui-Xiong Zhong:** writing – original draft, methodology, formal analysis, conceptualization. **Meng-Ying Wu:** writing – original draft, methodology, formal analysis, conceptualization. **Fu-Xiang Li:** formal analysis, data curation. **Xiang-Yu Chen:** formal analysis, data curation. **Meng-Yuan Jiang:** investigation, data curation. **Mei-Ting Zhang:** investigation, data curation. **An-Qi Li:** investigation, data curation. **Zi-Teng Yu:** investigation, data curation. **Wei Liu:** investigation, data curation. **Ke-Ke Cheng:** writing – review and editing, supervision, resources, methodology, project administration.

## Funding

No funding was received for this manuscript.

## Disclosure

It is the original work of the authors. All authors have agreed to submit the manuscript to the International Journal of Food Science.

## Ethics Statement

The authors have nothing to report.

## Conflicts of Interest

The authors declare no conflicts of interest.

## Supporting information


**Supporting Information** Additional supporting information can be found online in the Supporting Information section. Text S1: OPLS‐DA and PLSR were applied to a composite GC‐IMS and E‐tongue dataset using SIMCA 14.1, where PLSR identified critical variables with VIP > 1 for subsequent analysis. Table S1: Detailed information on green tea. Table S2: Method validation parameters for key aroma compounds. Table S3: Method validation parameters for amino acid analysis. Table S4: Method validation parameters for major phenolic compounds.

## Data Availability

The data that support the findings of this study are available on request from the corresponding author. The data are not publicly available due to privacy or ethical restrictions.

## References

[1] Aktumsek A. , Zengin G. , Guler G. O. , Cakmak Y. S. , and Duran A. , Assessment of the Antioxidant Potential and Fatty Acid Composition of Four *Centaurea* L. Taxa From Turkey, Food Chemistry. (2013) 141, no. 1, 91–97, 10.1016/j.foodchem.2013.02.092, 2-s2.0-84877090042, 23768332. 23768332

[2] Chen R. , Sun L. , Zhang S. , Li Q. , Wen S. , Lai X. , Li Q. , Cao J. , and Sun S. , Mechanisms and Quality Variations of Non-Volatile and Volatile Metabolites in Black Tea From Various Ages of Tea Trees: Insights From Metabolomics Analysis, Food Chemistry: X. (2024) 22, 101470, 10.1016/j.fochx.2024.101470. 38883921 PMC11176668

[3] Peng Q. , Li S. , Zheng H. , Meng K. , Jiang X. , Shen R. , Xue J. , and Xie G. , Characterization of Different Grades of Jiuqu Hongmei Tea Based on Flavor Profiles Using HS-SPME-GC-MS Combined With E-Nose and E-Tongue, Food Research International. (2023) 172, 113198, 10.1016/j.foodres.2023.113198. 37689946

[4] Wang Q. , Li M. , Wang J. , Ma X. , Liu L. , Wang P. , Hu J. , Zhang X. , and Qu F. , Exploring the Effect of Greenhouse Covering Cultivation on the Changes of Sensory Quality and Flavor Substances of Green Tea, Food Chemistry: X. (2024) 24, 101885, 10.1016/j.fochx.2024.101885. 39483358 PMC11525458

[5] Zhu Y. , Lv H. , Dai W. , Guo L. , Tan J. , Zhang Y. , Yu F. , Shao C. , Peng Q. , and Lin Z. , Separation of Aroma Components in Xihu Longjing Tea Using Simultaneous Distillation Extraction With Comprehensive Two-Dimensional Gas Chromatography-Time-of-Flight Mass Spectrometry, Separation and Purification Technology. (2016) 164, 146–154, 10.1016/j.seppur.2016.03.028, 2-s2.0-84961671411.

[6] Zhu M. , Li E. , and He H. , Determination of Volatile Chemical Constitutes in Tea by Simultaneous Distillation Extraction, Vacuum Hydrodistillation and Thermal Desorption, Chromatographia. (2008) 68, 603–610, 10.1365/s10337-008-0732-1, 2-s2.0-53549090142.

[7] Wang S. , Chen H. , and Sun B. , Recent Progress in Food Flavor Analysis Using Gas Chromatography–Ion Mobility Spectrometry (GC–IMS), Food Chemistry. (2020) 315, 126158, 10.1016/j.foodchem.2019.126158. 32014672

[8] Ho C. T. , Zheng X. , and Li S. , Tea Aroma Formation, Food Science and Human Wellness. (2015) 4, no. 1, 9–27, 10.1016/j.fshw.2015.04.001.

[9] Cheng L. , Wang Y. , Zhang J. , Zhu J. , Liu P. , Xu L. , Wei K. , Zhou H. , Peng L. , Zhang J. , Wei X. , and Liu Z. , Dynamic Changes of Metabolic Profile and Taste Quality During the Long-Term Aging of Qingzhuan Tea: The Impact of Storage Age, Food Chemistry. (2021) 359, 129953, 10.1016/j.foodchem.2021.129953. 34000695

[10] Türközü D. and Şanlier N. , L-Theanine, Unique Amino Acid of Tea, and Its Metabolism, Health Effects, and Safety, Critical Reviews in Food Science and Nutrition. (2017) 57, no. 8, 1681–1687, 10.1080/10408398.2015.1016141, 2-s2.0-85015145922. 26192072

[11] Williams J. L. , Everett J. M. , D’Cunha N. M. , Sergi D. , Georgousopoulou E. N. , Keegan R. J. , McKune A. J. , Mellor D. D. , Anstice N. , and Naumovski N. , The Effects of Green Tea Amino Acid L-Theanine Consumption on the Ability to Manage Stress and Anxiety Levels: A Systematic Review, Plant Foods for Human Nutrition. (2020) 75, no. 1, 12–23, 10.1007/s11130-019-00771-5, 31758301. 31758301

[12] Zhang L. , Zhang H. , Zhang X. , Tang H. , Ruan J. , Li C. , and Zhang Q. , Volatile and Flavour Compounds Analysis Reveal Quality Constituents of Qianlin Tea (*Camellia cuspidata*) to Diversify Tea Products, Applied Food Research. (2024) 4, no. 2, 100624, 10.1016/j.afres.2024.100624.

[13] Wu B. , He C. , Ma Y. , Shen J. , Zhang L. H. , Peng Y. , and Xiao P. G. , Investigation of Free Amino Acid, Total Phenolics, Antioxidant Activity And Purine Alkaloids To Assess The Health Properties Of Non-Camellia Tea, Acta Pharmaceutica Sinica B. (2016) 6, no. 2, 170–181, 10.1016/j.apsb.2015.11.003, 2-s2.0-84971571999. 27006902 PMC4788713

[14] Wang Y. , Deng G. , Huang L. , and Ning J. , Sensory-Directed Flavor Analysis Reveals the Improvement in Aroma Quality of Summer Green Tea by Osmanthus Scenting, Food Chemistry: X. (2024) 23, 101571, 10.1016/j.fochx.2024.101571. 39007121 PMC11239469

[15] Zhang Z. , Zhang Y. , Li J. , Fu C. , and Zhang X. , The Neuroprotective Effect of Tea Polyphenols on the Regulation of Intestinal Flora, Molecules. (2021) 26, no. 12, 10.3390/molecules26123692. PMC8233780 34204244

[16] Chowdhury A. , Sarkar J. , Chakraborti T. , Pramanik P. K. , and Chakraborti S. , Protective Role of Epigallocatechin-3-Gallate in Health and Disease: A Perspective, Biomedicine & Pharmacotherapy. (2016) 78, 50–59, 10.1016/j.biopha.2015.12.013, 2-s2.0-84958645581. 26898424

[17] Tang X. , Chen X. , Li F. , Huang M. , Xie L. , Ge J. , Ling H. , and Cheng K. , Analysis of Pickled Cucumber Products, Based on Microbial Diversity and Flavor Substance Detection, Foods. (2024) 13, no. 8, 10.3390/foods13081275. PMC11048978 38672946

[18] Yang X. , Zhang T. , Yang D. , and Xie J. , Application of Gas Chromatography-Ion Mobility Spectrometry in the Analysis of Food Volatile Components, Acta Chromatographica. (2022) 35, no. 1, 35–45, 10.1556/1326.2022.01005.

[19] Segura-Borrego M. P. , Martín-Gómez A. , Ríos-Reina R. , Cardador M. J. , Morales M. L. , Arce L. , and Callejón R. M. , A Non-Destructive Sampling Method for Food Authentication Using Gas Chromatography Coupled to Mass Spectrometry or Ion Mobility Spectrometry, Food Chemistry. (2022) 373, 131540, 10.1016/j.foodchem.2021.131540. 34799128

[20] Guo L. , Xie C. , Zhao F. , Zhang Y. , and Lin Z. , Comparison of Volatile Compounds Among Four Types of Teas Analyzed Using Gas Chromatography–Ion Mobility Spectrometry, Foods. (2024) 13, no. 13, 10.3390/foods13132043, 38998549. PMC11241802 38998549

[21] Kang H. J. , Yang H. J. , Kim M. J. , Han E. S. , and Kwon D. Y. , Metabolomic Analysis Of Meju During Fermentation By Ultra Performance Liquid Chromatography-Quadrupole-Time Of Flight Mass Spectrometry (UPLC-Q-TOF MS), Food Chemistry. (2011) 127, no. 3, 1056–1064, 10.1016/j.foodchem.2011.01.080, 2-s2.0-79952538972, 25214096. 25214096

[22] Wu R. , Yu M. , Liu X. , Meng L. , Wang Q. , Xue Y. , Wu J. , and Yue X. , Changes In Flavour And Microbial Diversity During Natural Fermentation Of Suan-Cai, A Traditional Food Made In Northeast China, International Journal of Food Microbiology. (2015) 211, 23–31, 10.1016/j.ijfoodmicro.2015.06.028, 2-s2.0-84936146211, 26159472. 26159472

[23] Zhang S. , Xia H. , Sun S. , Ma C. , Li F. , Pan S. , Zhang Z. , Lai X. , Li Q. , Hao M. , Sun L. , and Chen R. , Analysis of Volatile Metabolites and Differences in Aroma Quality of Green Tea, Black Tea and Oolong Tea Prepared From Highly Aromatic Tea Variety (Dancong), Food Research International. (2025) 221, Part 3, 117402, 10.1016/j.foodres.2025.117402, 41214914. 41214914

[24] Feng X. , Wang H. , Wang Z. , Huang P. , and Kan J. , Discrimination and Characterization of the Volatile Organic Compounds in Eight Kinds of Huajiao With Geographical Indication of China Using Electronic Nose, HS-GC-IMS and HS-SPME-GC-MS, Food chemistry. (2022) 375, 131671, 10.1016/j.foodchem.2021.131671, 34865919. 34865919

[25] Gerhardt N. , Birkenmeier M. , Schwolow S. , Rohn S. , and Weller P. , Volatile-Compound Fingerprinting by Headspace-Gas-Chromatography Ion-Mobility Spectrometry (HS-GC-IMS) as a Benchtop Alternative to ^1^H NMR Profiling for Assessment of the Authenticity of Honey, Analytical Chemistry. (2018) 90, no. 3, 1777–1785, 10.1021/acs.analchem.7b03748, 2-s2.0-85041425487. 29298045

[26] Xu J. , Zhang Y. , Hu C. , Yu B. , Wan C. , Chen B. , Lu L. , Yuan L. , Wu Z. , and Chen H. , The Flavor Substances Changes in Fuliang Green Tea During Storage Monitoring by GC–MS and GC-IMS, Food Chemistry: X. (2024) 21, 101047, 10.1016/j.fochx.2023.101047. 38187940 PMC10770431

[27] Vautz W. , Slodzynski R. , Hariharan C. , Seifert L. , Nolte J. , Fobbe R. , Sielemann S. , Lao B. C. , Huo R. , Thomas C. L. P. , and Hildebrand L. , Detection of Metabolites of Trapped Humans Using Ion Mobility Spectrometry Coupled With Gas Chromatography, Analytical Chemistry. (2013) 85, no. 4, 2135–2142, 10.1021/ac302752f, 2-s2.0-84874045766. 23249433

[28] Cumeras R. , Figueras E. , Davis C. E. , Baumbach J. I. , and Gràcia I. , Review on Ion Mobility Spectrometry. Part 1: Current Instrumentation, Analyst. (2015) 140, no. 5, 1376–1390, 10.1039/C4AN01100G, 2-s2.0-84923206701. 25465076 PMC4331213

[29] Creaser C. S. , Griffiths J. R. , Bramwell C. J. , Noreen S. , Hill C. A. , and Thomas C. L. P. , Ion Mobility Spectrometry: A Review. Part 1. Structural Analysis by Mobility Measurement, Analyst. (2004) 129, no. 11, 10.1039/b404531a, 2-s2.0-9144267429.

[30] Polat S. , Color Quality, Ascorbic Acid, Total Carotenoid, and Volatile Compounds of Dried Orange Slices as Influenced by Packaging Methods and Storage Conditions, Journal of Food Processing and Preservation. (2022) 46, no. 6, 10.1111/jfpp.15898.

[31] Tekgül Y. and Baysal T. , Optimization of Process Conditions for Vacuum Microwave Drying of Lemon Peel by Response Surface Methodology: Quality Characteristics and Volatile Compounds, Journal of Food Process Engineering. (2019) 42, no. 5, 13080, 10.1111/jfpe.13080, 2-s2.0-85065211507.

[32] Shumin Y. , Shaomin W. , Yongsheng Z. , Zhongmin L. , and Meizhen T. , Analysis of Flavor Components in HS-GC-IMS and Antioxidant Properties of Black *Lycium barbarum* Rice Wine, Journal of Food and Nutrition Research. (2021) 9, no. 1, 18–25, 10.12691/jfnr-9-1-3.

[33] Meng J. , Liu Z. , Gou C.-L. , Rogers K. M. , Yu W.-J. , Zhang S.-S. , Yuan Y.-W. , and Zhang L. , Geographical Origin of Chinese Wolfberry (Goji) Determined by Carbon Isotope Analysis of Specific Volatile Compounds, Journal of Chromatography B. (2019) 1105, 104–112, 10.1016/j.jchromb.2018.12.011, 2-s2.0-85058637697. 30580182

[34] Niu M. , Huang J. , Jin Y. , Wu C. , and Zhou R. , Volatiles and Antioxidant Activity of Fermented Goji (LyciumChinese) Wine: Effect of Different Oak Matrix (Barrel, Shavings and Chips), International Journal of Food Properties. (2017) 9, 1–13, 10.1080/10942912.2017.1362649, 2-s2.0-85039555154.

[35] Zhu Y. , Lv H. P. , Shao C. Y. , Kang S. , Zhang Y. , Guo L. , Dai W. D. , Tan J. F. , Peng Q. H. , and Lin Z. , Identification of Key Odorants Responsible for Chestnut-Like Aroma Quality of Green Teas, Food Research International. (2018) 108, 74–82, 10.1016/j.foodres.2018.03.026, 2-s2.0-85044003326, 29735103. 29735103

[36] Yang Y. , Zhang M. , Hua J. , Deng Y. , Jiang Y. , Li J. , Wang J. , Yuan H. , and Dong C. , Quantitation of Pyrazines in Roasted Green Tea by Infrared-Assisted Extraction Coupled to Headspace Solid-Phase Microextraction in Combination With GC-QqQ-MS/MS, Food Research International. (2020) 134, 109167, 10.1016/j.foodres.2020.109167. 32517930

[37] Diez-Simon C. , Eichelsheim C. , Mumm R. , and Hall R. D. , Chemical and Sensory Characteristics of Soy Sauce: A Review, Journal of Agricultural and Food Chemistry. (2020) 68, no. 42, 11612–11630, 10.1021/acs.jafc.0c04274, 32880168. 32880168 PMC7581291

[38] Pan X. , Bi S. , Lao F. , and Wu J. , Factors Affecting Aroma Compounds in Orange Juice and Their Sensory Perception: A Review, Food Research International. (2023) 169, 112835, 10.1016/j.foodres.2023.112835, 37254409. 37254409

[39] Flaig M. , Qi S. , Wei G. , Yang X. , and Schieberle P. , Characterization of the Key Odorants in a High-Grade Chinese Green Tea Beverage (*Camellia sinensis; Jingshan cha*) by Means of the Sensomics Approach and Elucidation of Odorant Changes in Tea Leaves Caused by the Tea Manufacturing Process, Journal of Agricultural and Food Chemistry. (2020) 68, no. 18, 5168–5179, 10.1021/acs.jafc.0c01300. 32251584

[40] Ge S. , Chen Y. , Ding S. , Zhou H. , Jiang L. , Yi Y. , Deng F. , and Wang R. , Changes in Volatile Flavor Compounds of Peppers During Hot Air Drying Process Based on Headspace-Gas Chromatography-Ion Mobility Spectrometry (HS-GC-IMS), Journal of the Science of Food and Agriculture. (2020) 100, no. 7, 3087–3098, 10.1002/jsfa.10341. 32083310

[41] Yang C. , Wang Z. , Xu M. , Wei K. , Dai Q. , Wan X. , Leong O. , Lin R. , Cui C. , and Hou R. , The Chemical Basis of Aroma/Taste and Color Formation in Green Tea Infusion During Cold Brewing Revealed by Metabolomics Analysis, Food Chemistry. (2025) 479, 143788, 10.1016/j.foodchem.2025.143788, 40073559. 40073559

[42] Li L. , Wen M. , Hu W. , Huang X. , Li W. , Han Z. , and Zhang L. , Non-Volatile Metabolite and In Vitro Bioactivity Differences in Green, White, and Black Teas, Food Chemistry. (2025) 477, 143580, 10.1016/j.foodchem.2025.143580. 40031135

[43] Chen Q. , Zhu Y. , Liu Y. , Liu Y. , Dong C. , Lin Z. , and Teng J. , Black Tea Aroma Formation During the Fermentation Period, Food Chemistry. (2022) 374, 131640, 10.1016/j.foodchem.2021.131640, 34839968. 34839968

[44] Guo M. , Chen Z. , Ding Z. , Wang D. , Qi D. , Lu M. , Wang M. , and Dong C. , Traceability of Rizhao Green Tea Origin Based on Multispectral Data Fusion Strategy and Chemometrics, Food Chemistry: X. (2025) 27, 102346, 10.1016/j.fochx.2025.102346. 40213336 PMC11982953

[45] Li Y. , Han Z. , Wang M. , Yan Y. , Ma R. , Wang H. , and Deng W. W. , Metabolomics and Sensory Evaluation Reveal the Influence of Four Albino Tea Cultivars on the Quality of Processed Green Tea, Food Research International. (2025) 209, 116180, 10.1016/j.foodres.2025.116180, 40253163. 40253163

[46] Yang P. , Song H. , Wang L. , and Jing H. , Characterization of Key Aroma-Active Compounds in Black Garlic by Sensory-Directed Flavor Analysis, Journal of Agricultural and Food Chemistry. (2019) 67, no. 28, 7926–7934, 10.1021/acs.jafc.9b03269, 2-s2.0-85069833553. 31250635

[47] Yang X. , Zhu K. , Guo H. , Geng Y. , Lv W. , Wang S. , Guo Y. , Qin P. , and Ren G. , Characterization of Volatile Compounds in Differently Coloured Chenopodium quinoa Seeds Before and After Cooking by Headspace-Gas Chromatography-Ion Mobility Spectrometry, Food Chemistry. (2021) 348, 129086, 10.1016/j.foodchem.2021.129086, 33508608. 33508608

[48] Qi H. , Ding S. , Pan Z. , Li X. , and Fu F. , Characteristic Volatile Fingerprints and Odor Activity Values in Different Citrus-Tea by HS-GC-IMS and HS-SPME-GC-MS, Molecules (Basel, Switzerland). (2020) 25, no. 24, 10.3390/molecules25246027. PMC7766395 33352716

[49] Sereshti H. , Samadi S. , and Jalali-Heravi M. , Determination of Volatile Components of Green, Black, Oolong and White Tea by Optimized Ultrasound-Assisted Extraction-Dispersive Liquid–Liquid Microextraction Coupled With Gas Chromatography, Journal of Chromatography A. (2013) 1280, 1–8, 10.1016/j.chroma.2013.01.029, 2-s2.0-84873523422. 23375769

[50] Jiang H. , Zhang M. , Bhandari B. , and Adhikari B. , Application of Electronic Tongue for Fresh Foods Quality Evaluation: A Review, Food Reviews International. (2018) 34, no. 8, 746–769, 10.1080/87559129.2018.1424184, 2-s2.0-85053334742.

[51] An X. , Wang Z. , Li J. , Nie X. , Liu K. , Zhang Y. , and Ao C. , Analysis of Flavor-Related Compounds in Fermented Persimmon Beverages Stored at Different Temperatures, LWT. (2022) 163, 113524, 10.1016/j.lwt.2022.113524.

[52] Dai W. , Lou N. , Xie D. , Hu Z. , Song H. , Lu M. , Shang D. , Wu W. , Peng J. , Yin P. , and Lin Z. , *N*-Ethyl-2-Pyrrolidinone-Substituted Flavan-3-Ols With Anti-Inflammatory Activity in Lipopolysaccharide-Stimulated Macrophages Are Storage-Related Marker Compounds for Green Tea, Journal of Agricultural and Food Chemistry. (2020) 68, no. 43, 12164–12172, 10.1021/acs.jafc.0c03952. 33074673

[53] Chen Q. , Shi J. , Mu B. , Chen Z. , Dai W. , and Lin Z. , Metabolomics Combined With Proteomics Provides a Novel Interpretation of the Changes in Nonvolatile Compounds During White Tea Processing, Food Chemistry. (2020) 332, 127412, 10.1016/j.foodchem.2020.127412, 32623128. 32623128

[54] Kaneko S. , Kumazawa K. , Masuda H. , Henze A. , and Hofmann T. , Molecular and Sensory Studies on the Umami Taste of Japanese Green Tea, Journal of Agricultural and Food Chemistry. (2006) 54, no. 7, 2688–2694, 10.1021/jf0525232, 2-s2.0-33645983618. 16569062

[55] Zhang L. , Cao Q. Q. , Granato D. , Xu Y. Q. , and Ho C. T. , Association Between Chemistry and Taste of Tea: A Review, Trends in Food Science & Technology. (2020) 101, 139–149, 10.1016/j.tifs.2020.05.015.

[56] Dai W. , Qi D. , Yang T. , Lv H. , Guo L. , Zhang Y. , Zhu Y. , Peng Q. , Xie D. , Tan J. , and Lin Z. , Nontargeted Analysis Using Ultraperformance Liquid Chromatography–Quadrupole Time-of-Flight Mass Spectrometry Uncovers the Effects of Harvest Season on the Metabolites and Taste Quality of Tea (*Camellia sinensis* L.), Journal of Agricultural and Food Chemistry. (2015) 63, no. 44, 9869–9878, 10.1021/acs.jafc.5b03967, 2-s2.0-84946949657. 26494158

[57] Trisha A. T. , Shakil M. H. , Talukdar S. , Rovina K. , Huda N. , and Zzaman W. , Tea Polyphenols and Their Preventive Measures Against Cancer: Current Trends and Directions, Foods (Basel, Switzerland). (2022) 11, no. 21, 10.3390/foods11213349. PMC9658101 36359962

[58] Yu H. , Wang X. , Xie J. , Ai L. , Chen C. , and Tian H. , Isolation and Identification of Bitter-Tasting Peptides in Shaoxing Rice Wine Using Ultra-Performance Liquid Chromatography Quadrupole Time-of-Flight Mass Spectrometry Combined With Taste Orientation Strategy, Journal of Chromatography. A. (2022) 1676, 463193, 10.1016/j.chroma.2022.463193. 35709603

[59] Zhao S. , Niu C. , Yang X. , Xu X. , Zheng F. , Liu C. , Wang J. , and Li Q. , Roles of Sunlight Exposure on Chemosensory Characteristic of Broad Bean Paste by Untargeted Profiling of Volatile Flavors and Multivariate Statistical Analysis, Food Chemistry. (2022) 381, 132115, 10.1016/j.foodchem.2022.132115. 35124490

[60] Chou O. , Ali A. , Subbiah V. , Barrow C. J. , Dunshea F. R. , and Suleria H. A. R. , LC-ESI-QTOF-MS/MS Characterisation of Phenolics in Herbal Tea Infusion and Their Antioxidant Potential, Fermentation. (2021) 7, no. 2, 10.3390/fermentation7020073.

[61] Lorenz M. , Lehmann S. , Djordjevic I. , Düsterhöft T. , Zimmermann B. F. , Stangl K. , and Stangl V. , Vasodilation of Tea Polyphenols Ex Vivo Is Mediated by Hydrogen Peroxide Under Rapid Compound Decay, Antioxidants. (2020) 9, no. 5, 10.3390/antiox9050390. PMC7278881 32392754

[62] Wang D. , Wang T. , Li Z. , Guo Y. , and Granato D. , Green Tea Polyphenols Upregulate the Nrf2 Signaling Pathway and Suppress Oxidative Stress and Inflammation Markers in D-Galactose-Induced Liver Aging in Mice, Frontiers in Nutrition. (2022) 9, 836112, 10.3389/fnut.2022.836112. 35284456 PMC8904921

[63] Yang L. , Lu Z. , Lu J. , and Wu D. , Evaluation of the Antioxidant Characteristics of Craft Beer With Green Tea, Journal of Food Science. (2023) 88, no. 2, 625–637, 10.1111/1750-3841.16441. 36576119

[64] Zhang J. Y. , Cui H. C. , Feng Z. H. , Wang W. W. , Zhao Y. , Deng Y. L. , Jiang H. Y. , Yin J. F. , and Engelhardt U. H. , Bitterness Quantification and Simulated Taste Mechanism of Theasinensin A From Tea, Frontiers in Nutrition. (2023) 10, 1138023, 10.3389/fnut.2023.1138023, 37229471. 37229471 PMC10203438

[65] Yu Z. and Yang Z. , Understanding Different Regulatory Mechanisms of Proteinaceous and Non-Proteinaceous Amino Acid Formation in Tea (Camellia sinensis) Provides New Insights Into the Safe and Effective Alteration of Tea Flavor and Function, Critical Reviews in Food Science and Nutrition. (2020) 60, no. 5, 844–858, 10.1080/10408398.2018.1552245. 30614265

[66] Liu Z. , Ran Q. , Li Q. , Yang T. , Dai Y. , Zhang T. , Fang S. , Pan K. , and Long L. , Interaction Between Major Catechins and Umami Amino Acids in Green Tea Based on Electronic Tongue Technology, Journal of Food Science. (2023) 88, no. 6, 2339–2352, 10.1111/1750-3841.16543. 37138542

[67] Sun L. , Wen S. , Zhang S. , Li Q. , Cao J. , Chen R. , Chen Z. , Zhang Z. , Li Z. , Li Q. , Lai Z. , and Sun S. , Study on Flavor Quality Formation in Green and Yellow Tea Processing by Means of UPLC-MS Approach, Food Chemistry: X. (2024) 22, 101342, 10.1016/j.fochx.2024.101342. 38665631 PMC11043817

[68] El-Saadony M. T. , Yang T. , Saad A. M. , Alkafaas S. S. , Elkafas S. S. , Eldeeb G. S. , Mohammed D. M. , Salem H. M. , Korma S. A. , Loutfy S. A. , Alshahran M. Y. , Ahmed A. E. , Mosa W. F. A. , Abd El-Mageed T. A. , Ahmed A. F. , Fahmy M. A. , El-Tarabily M. K. , Mahmoud R. M. , AbuQamar S. F. , El-Tarabily K. A. , and Lorenzo J. M. , Polyphenols: Chemistry, Bioavailability, Bioactivity, Nutritional Aspects and Human Health Benefits: A Review, International Journal of Biological Macromolecules. (2024) 277, Part 3, 134223, 10.1016/j.ijbiomac.2024.134223. 39084416

[69] Shil A. , Banerjee A. , Roy J. , Pal M. , Das D. , Paul R. , Maji B. K. , and Sikdar M. , The Potential Antibacterial Effects of Tea Polyphenols, Drug Metabolism and Personalized Therapy. (2024) 39, no. 3, 103–114, 10.1515/dmpt-2024-0058. 39263725

[70] Shirakami Y. and Shimizu M. , Possible Mechanisms of Green Tea and Its Constituents Against Cancer, Molecules (Basel, Switzerland). (2018) 23, no. 9, 10.3390/molecules23092284, 2-s2.0-85052865539. PMC6225266 30205425

[71] Zhao Y. and Zhao B. , Protection of Green Tea Polyphenols Against Neurodegenerative Diseases: Evidence and Possible Mechanisms, Journal of Nutrition. (2025) 155, no. 4, 1077–1088, 10.1016/j.tjnut.2025.02.010, 39956389. 39956389

[72] Liang J. F. , Wu H. L. , Lu M. F. , and Li Y. , HS-SPME-GC-MS Untargeted Metabolomics Reveals Key Volatile Compound Changes During Liupao Tea Fermentation, Food Chemistry. (2024) 23, 101764, 10.1016/j.fochx.2024.101764. PMC11401112 39280217

[73] Hu J. , Xie J. , Wang Q. , Tang J. , Zhou X. , Yuan H. , Jiang Y. , and Yang Y. , Unraveling the Dynamic Variations of Volatile and Non-Volatile Metabolites in Green Tea During the Yellow-Light Irradiation Spreading Process by Targeted and Untargeted Metabolomics, LWT. (2025) 215, 117152, 10.1016/j.lwt.2024.117152.

[74] Jiao Y. , Cai M. , Zhang X. , Feng Z. , Zhang Q. , Li L. , Jin G. , Fan S. , and Lu L. , Impact of Spreading Time on Flavor Quality in Duyun Maojian Summer Green Tea, LWT. (2024) 214, 117103, 10.1016/j.lwt.2024.117103.

[75] Zhou H. , Liu Y. , Wu Q. , Zhang X. , Wang H. , and Lei P. , The Manufacturing Process Provides Green Teas With Differentiated Nonvolatile Profiles and Influences the Deterioration of Flavor During Storage at Room Temperature, Food Chemistry: X. (2024) 22, 101371, 10.1016/j.fochx.2024.101371, 38633742. 38633742 PMC11021834

[76] Jin G. , Li T. , Yang C. , Du X. , Dai Q. , Li A. , and Hou R. , Deciphering the Aromatic Signature of Taiping Houkui Premium Green Tea: The Role of Key Floral Compounds and Process Control on Volatile Compounds, LWT. (2024) 212, 116970, 10.1016/j.lwt.2024.116970.

[77] Huang S. , Tao L. , Xu L. , Shu M. , Qiao D. , Wen H. , Xie H. , Chen H. , Liu S. , Xie D. , Wei C. , and Zhu J. , Discrepancy on the Flavor Compound Affect the Quality of Taiping Houkui Tea From Different Production Regions, Food Chemistry: X. (2024) 23, 101547, 10.1016/j.fochx.2024.101547. 38974194 PMC11225684

[78] Qiao D. , Zhu J. , Mi X. , Xie H. , Shu M. , Chen M. , Li R. , Liu S. , and Wei C. , Effects of Withering Time of Fresh Leaves on the Formation of Flavor Quality of Taiping Houkui Tea, LWT. (2023) 182, 114833, 10.1016/j.lwt.2023.114833.

[79] Xin X. L. , Fu W. J. , Chen Y. , Zhou R. F. , Sun W. Q. , Ding B. M. , Peng X. T. , and Gu H. W. , GC-MS-Based Untargeted Metabolomics Reveals The Key Volatile Organic Compounds For Discriminating Grades Of Yichang Big-Leaf Green Tea, LWT - Food Science and Technology. (2022) 171, 114148, 10.1016/j.lwt.2022.114148.

[80] Yang Y. , Wang B. , Fu Y. , Shi Y. , Chen F. , Guan H. , Liu L. , Zhang C. , Zhu P. , Liu Y. , and Zhang N. , HS-GC-IMS With PCA to Analyze Volatile Flavor Compounds Across Different Production Stages of Fermented Soybean Whey Tofu, Food Chemistry. (2021) 346, 128880, 10.1016/j.foodchem.2020.128880. 33418415

[81] Zhao T. , Li C. , Wang S. , and Song X. , Green Tea (*Camellia sinensis*): A Review of Its Phytochemistry, Pharmacology, and Toxicology, Molecules (Basel, Switzerland). (2022) 27, no. 12, 10.3390/molecules27123909, 35745040. PMC9231383 35745040

[82] Zhou L. , Ma Y. , Xu J. , Hu Y. , Zhao M. , Marchioni E. , and Fu H. , Determination and Comparison of Lipid Profiles of Chinese Green Tea Varieties Using Untargeted Lipidomics Analysis Combined With Chemometrics, Food Chemistry. (2025) 477, 143467, 10.1016/j.foodchem.2025.143467. 39999551

