# Supplementary material for: Guizhou Green Tea Metabolomics Analysis: Volatile Metabolites Based on HS‐GC‐IMS and UPLC‐Q‐TOF‐MSMS and Quality Evaluation
Source: Int J Food Sci. 2026 May 18;2026:1031648. doi: 10.1155/ijfo/1031648 (PMC13184546; doi:10.1155/ijfo/1031648)
Supplement: Supplementary file 1 — Supporting Information Additional supporting information can be found online in the Supporting Information section. Text S1: OPLS‐DA and PLSR were applied to a composite GC‐IMS and E‐tongue dataset using SIMCA 14.1, where PLSR identified critical variables with VIP > 1 for subsequent analysis. Table S1: Detailed information on green tea. Table S2: Method validation parameters for key aroma compounds. Table S3: Method validation parameters for amino acid analysis. Table S4: Method validation parameters for major phenolic compounds. [file IJFO-2026-1031648-s001.docx]

**Test S1.**

In the present study, OPLS-DA and PLSR methodologies were employed for a composite dataset derived from GC-IMS and E-tongue analyses, utilizing the SIMCA 14.1 software package (Umetrics AB, Umea, Sweden). For quantitative analysis, an initial variable screening was conducted via PLSR, where variables with a VIP greater than one were identified as critical and were thus selected for further analytical progression.

**Table S1.** Detailed information on green tea

| **Count** | **Sample name** | **Manufacturer** |
| --- | --- | --- |
| **1** | Mei Tan Cui Ya-1 | Lanxin Industry Co., LTD. Guizhou, China |
| **2** | Mei Tan Cui Ya-2 | Lanxin Industry Co., LTD. Guizhou, China |
| **3** | Mei Tan Cui Ya-3 | Lanxin Industry Co., LTD. Guizhou, China |
| **4** | Mei Tan Mao Feng-1 | Fuying Food Development Industry Co., LTD. Guizhou, China |
| **5** | Mei Tan Mao Feng-2 | Fuying Food Development Industry Co., LTD. Guizhou, China |
| **6** | Mei Tan Mao Feng-3 | Fuying Food Development Industry Co., LTD. Guizhou, China |
| **7** | Dou Yun Mao Jian-1 | Maojian Tea Development Industry Co., LTD. Guizhou, China |
| **8** | Dou Yun Mao Jian-2 | Maojian Tea Development Industry Co., LTD. Guizhou, China |
| **9** | Dou Yun Mao Jian-3 | Maojian Tea Development Industry Co., LTD. Guizhou, China |

**Table S2.** Method validation parameters for key aroma compounds. (Recovery was determined at three spike levels (low, medium, high); values represent mean ± SD. RSD: Relative standard deviation.)

| Compound | Linear Range (ng/g) | R² | LOD (ng/g) | LOQ (ng/g) | Recovery (%) | Intra-day RSD (%) | Inter-day RSD (%) |
| --- | --- | --- | --- | --- | --- | --- | --- |
| Heptanal | 0.5-200 | 0.9982 | 0.15 | 0.50 | 92.3±4.2 | 3.8 | 5.2 |
| Butanol | 1.0-500 | 0.9975 | 0.30 | 1.00 | 95.1±3.8 | 4.1 | 6.3 |
| Pentanol | 0.5-300 | 0.9989 | 0.12 | 0.40 | 89.7±5.1 | 3.5 | 4.8 |
| 2-Hexanol | 0.2-100 | 0.9968 | 0.08 | 0.25 | 93.5±3.2 | 2.9 | 4.1 |
| 2-Pentanol | 0.5-150 | 0.9974 | 0.10 | 0.35 | 91.2±4.5 | 3.2 | 5.6 |
| Propanol | 0.5-200 | 0.9981 | 0.15 | 0.50 | 94.8±3.6 | 3.9 | 5.8 |
| Methyl 3-methylbutanoate | 0.1-80 | 0.9987 | 0.03 | 0.10 | 96.2±2.8 | 2.5 | 3.9 |
| 2-Furanmethanol acetate | 0.2-100 | 0.9979 | 0.05 | 0.18 | 88.9±4.8 | 4.2 | 6.1 |
| n-Propyl acetate | 0.5-200 | 0.9983 | 0.12 | 0.40 | 92.7±3.4 | 3.1 | 4.5 |
| Methyl-2-furoate | 0.05-50 | 0.9991 | 0.02 | 0.06 | 94.5±2.9 | 2.8 | 4.2 |
| Methyl acetate | 0.5-150 | 0.9976 | 0.15 | 0.50 | 91.8±4.1 | 3.6 | 5.3 |
| (E)-2-Pentenal | 0.2-100 | 0.9984 | 0.06 | 0.20 | 93.1±3.7 | 3.3 | 4.9 |
| 2-Phenylethanal | 0.1-80 | 0.9986 | 0.04 | 0.12 | 95.3±3.1 | 2.9 | 4.3 |
| 2,4-Heptadienal | 0.05-50 | 0.9992 | 0.02 | 0.05 | 92.8±3.5 | 3.1 | 4.6 |
| 2-Hexenal | 0.5-200 | 0.9978 | 0.15 | 0.50 | 90.5±4.3 | 3.8 | 5.7 |
| 6-Methyl-5-hepten-2-one | 0.2-100 | 0.9985 | 0.06 | 0.20 | 94.1±3.2 | 2.7 | 4.1 |
| 1-Penten-3-one | 0.1-80 | 0.9988 | 0.03 | 0.10 | 91.6±3.9 | 3.4 | 5.2 |
| 2-Butanone | 0.5-300 | 0.9971 | 0.20 | 0.65 | 93.4±4.0 | 3.6 | 5.4 |
| 5-Methyl-3-heptanone | 0.2-100 | 0.9982 | 0.05 | 0.18 | 92.2±3.6 | 3.2 | 4.8 |
| 2-Hexanone | 0.5-200 | 0.9979 | 0.12 | 0.40 | 89.8±4.4 | 3.9 | 5.9 |
| 3-Methyl-1-butanol | 0.1-100 | 0.9987 | 0.04 | 0.12 | 95.7±2.7 | 2.6 | 3.8 |
| trans-3-Hexenol | 0.5-200 | 0.9980 | 0.15 | 0.50 | 91.3±3.8 | 3.4 | 5.1 |

**Table S3.** Method Validation Parameters for Amino Acid Analysis.

| Amino Acid | Linear Range (μg/kg) | R² | LOD (μg/kg) | LOQ (μg/kg) | Recovery (%) | Intra-day RSD (%) | Inter-day RSD (%) |
| --- | --- | --- | --- | --- | --- | --- | --- |
| Glutamic acid | 10-1000 | 0.9991 | 0.05 | 0.15 | 98.2±3.2 | 2.8 | 4.1 |
| Theanine | 10-1000 | 0.9989 | 0.08 | 0.25 | 96.5±3.8 | 3.2 | 4.8 |
| Proline | 5-500 | 0.9993 | 0.03 | 0.10 | 95.8±2.9 | 2.5 | 3.9 |
| Valine | 5-500 | 0.9987 | 0.04 | 0.12 | 94.2±3.4 | 3.1 | 4.5 |
| Lysine | 5-500 | 0.9990 | 0.05 | 0.15 | 93.7±3.6 | 3.3 | 4.7 |
| Aspartic acid | 10-1000 | 0.9985 | 0.10 | 0.30 | 92.8±4.1 | 3.8 | 5.2 |
| Threonine | 5-500 | 0.9992 | 0.03 | 0.10 | 95.1±3.0 | 2.7 | 4.0 |

**Table S4.** Method validation parameters for major phenolic compounds. (These 15 compounds were quantified using authentic standards. Remaining phenolic compounds were semi-quantified using class-appropriate internal standards as described in Methods Section)

| Compound | Class | Linear Range (μg/g) | R² | LOD (μg/g) | LOQ (μg/g) | Recovery (%) | RSD (%) |
| --- | --- | --- | --- | --- | --- | --- | --- |
| Gallic acid | Phenolic acid | 0.1-100 | 0.9995 | 0.01 | 0.03 | 95.2±2.8 | 3.2 |
| Chlorogenic acid | Phenolic acid | 0.5-200 | 0.9991 | 0.02 | 0.06 | 93.8±3.1 | 3.5 |
| Caffeic acid | Phenolic acid | 0.1-50 | 0.9989 | 0.01 | 0.03 | 92.5±3.4 | 3.8 |
| Catechin | Flavanol | 1.0-500 | 0.9993 | 0.05 | 0.15 | 94.5±2.9 | 3.1 |
| Epicatechin | Flavanol | 0.5-200 | 0.9990 | 0.03 | 0.10 | 92.1±3.2 | 3.4 |
| Epicatechin gallate | Flavanol | 0.2-100 | 0.9987 | 0.02 | 0.06 | 91.8±3.5 | 3.7 |
| Quercetin | Flavonol | 0.1-50 | 0.9992 | 0.01 | 0.03 | 96.3±2.6 | 2.8 |
| Kaempferol | Flavonol | 0.1-50 | 0.9994 | 0.01 | 0.03 | 94.7±2.8 | 3.0 |
| Rutin | Flavonoid | 0.5-200 | 0.9988 | 0.02 | 0.08 | 93.2±3.3 | 3.6 |
| Myricetin | Flavonol | 0.1-50 | 0.9991 | 0.01 | 0.04 | 95.8±2.7 | 2.9 |
| Hyperoside | Flavonoid | 0.2-100 | 0.9986 | 0.02 | 0.06 | 92.4±3.4 | 3.7 |
| Procyanidin B2 | Flavanol | 0.5-200 | 0.9989 | 0.03 | 0.10 | 91.5±3.8 | 4.1 |
| Gallocatechin | Flavanol | 0.5-200 | 0.9990 | 0.03 | 0.10 | 93.1±3.1 | 3.3 |
| Vanillic acid | Phenolic acid | 0.1-50 | 0.9993 | 0.01 | 0.03 | 94.2±2.9 | 3.1 |
| p-Coumaric acid | Phenolic acid | 0.1-50 | 0.9991 | 0.01 | 0.04 | 92.8±3.2 | 3.4 |
